# Multimetallic Permethylpentalene Hydride Complexes

**DOI:** 10.1021/acs.inorgchem.2c01267

**Published:** 2022-07-25

**Authors:** Duncan A. X. Fraser, Zoë R. Turner, Robert T. Cooper, Jean-Charles Buffet, Jennifer C. Green, Dermot O’Hare

**Affiliations:** Department of Chemistry, Chemistry Research Laboratory, 12 Mansfield Road, Oxford OX1 3TA, U.K.

## Abstract

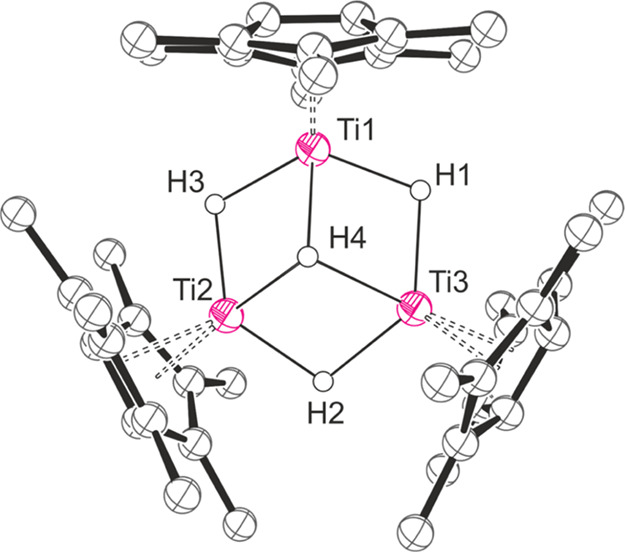

The synthesis and characterization of group 4 permethylpentalene (Pn* = C_8_Me_6_) hydride complexes are explored; in all cases, multimetallic hydride clusters were obtained. Group 4 lithium metal hydride clusters were obtained when reacting the metal dihalides with hydride transfer reagents such as LiAlH_4_, and these species featured an unusual hexagonal bipyramidal structural motif. Only the zirconium analogue was found to undergo hydride exchange in the presence of deuterium. In contrast, a trimetallic titanium hydride cluster was isolated on reaction of the titanium dialkyl with hydrogen. This diamagnetic, mixed valence species was characterized in the solid state, as well as by solution electron paramagnetic resonance and nuclear magnetic resonance spectroscopy. The structure was further probed and corroborated by density functional theory calculations, which illustrated the formation of a metal-cluster bonding orbital responsible for the diamagnetism of the complex. These permethylpentalene hydride complexes have divergent structural motifs and reactivity in comparison with related classical cyclopentadienyl analogues.

## Abstract 2

The synthesis and characterization of group 4 permethylpentalene (Pn* = C_8_Me_6_) hydride complexes are explored.

## Introduction

Transition metal hydride complexes play a key role in the addition of hydrogen to unsaturated substrates, one of the most common chemical transformations employed in the production of commodity and fine chemicals alike.^1^ The ability of metal hydrides to overcome kinetic barriers to hydrogen transfer has encouraged widespread investigation into their fundamental organometallic chemistry^2^ and role in homogeneous catalysis.^3^

Group 4 metal hydride complexes, typically metallocene hydrides,^4^ have been extensively explored and demonstrate a wide range of uses in the hydrogenation of olefinic bonds,^5^ polymerization catalysis,^6^ and the reduction of carbon oxides,^7^ as well as playing a role in understanding activation of dinitrogen.^8^ Notably, Schwartz’s reagent (Cp_2_Zr(*H*)Cl) has proved to be a powerful tool in organic synthesis, allowing for hydrozirconation and subsequent chemoselective C–C, C–N, and C–X bond formation with good functional group tolerance.^9^ Related polyhydride complexes not only possess interesting structural motifs but have also shown to demonstrate remarkable power in activating challenging bonds;^10^ Hou and co-workers have reported both dinitrogen reduction and cleavage^10c^ and C–C activation of benzene^10b^ by the trimetallic heptahydride cluster {(η^5^-C_5_Me_4_SiMe_3_)Ti}_3_(μ_3_-H)(μ_2_-H)_6_. Noncyclopentadienyl-supported group 4 metal hydrides are comparatively rarer;^11^ Okuda and co-workers have reported the preparation of [{Zr(Me_3_TACD)(μ-H)_2_}_2_][A]_2_ (Me_3_TACD = 1,4,7-trimethyl-1,4,7,10-tetraazacyclododecane; A = Al{OC(CF_3_)_3_}_4_, B{3,5-C_6_H_3_(CF_3_)_2_}_4_, B(3,5-C_6_H_3_Cl_2_)_4_, and BPh_4_) by hydrogenolysis (50 bar H_2_) of the corresponding neosilyl complexes.^11a^ Related hydrogenolysis with P_2_N_2_ Hf methyl complexes, {PhP(CH_2_SiMe_2_NSiMe_2_CH_2_)_2_PPh}HfMe_2_, reported by Fryzuk and co-workers, also afforded binuclear tetrahydride complexes.^11e^

There are a number of synthetic strategies used to access metal-hydride complexes. With reduced metal complexes, oxidative addition of H_2_ provides an atom economical route to install hydride ligands,^7a,12^ or alternatively alkyl, benzyl, or aryl derivatives can undergo σ-bond metathesis, which proceeds with loss of the protonated ligand.^13^ With metal-halide complexes, hydride transfer reagents can be employed,^4a,4c,14^ which provide a useful lever to control the reaction outcome by tuning the hydride donor capacity of the H^–^ source.^15^ Less commonly, reaction of metal-halide complexes with alkylating agents bearing β-hydrogens can allow for the synthesis of metal-hydride complexes following elimination.^16^

Herein, we describe two new group 4 lithium multimetallic hydride complexes, which are synthesized by the reaction of hydride transfer reagents and permethylpentalene (Pn* = C_8_Me_6_) metal-chloride precursors. In addition, the trimetallic titanium complex Pn*_3_Ti_3_(μ_2_-H)_3_(μ_3_-H) is isolated and its structure and bonding are investigated through spectroscopic studies as well as density functional theory calculations.

## Results and Discussion

### Synthesis of Group 4 Lithium Metal Hydride Clusters: Pn*_2_M_2_(μ_2_-H)_5_Li.thf_*x*_

**Scheme 1:**
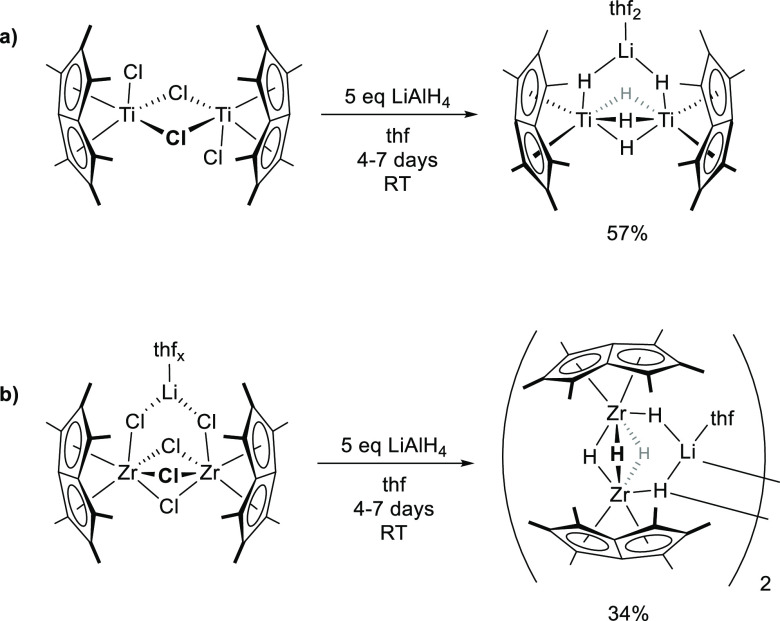
Synthesis of (a) Pn*_2_Ti_2_(μ_2_-H)_5_Li.thf_2_ and (b) [Pn*_2_Zr_2_(μ_2_-H)_4_(μ_3_-H)Li.thf]_2_

The reactivity of (Pn*TiCl)_2_(μ-Cl)_2_ with a variety of hydride transfer reagents was investigated. LiH, KH, and NaBH_4_ all displayed no reactivity in polar and nonpolar solvents at both ambient and elevated temperatures. By contrast LiAlH_4_ in thf was observed to successfully form hydride derivatives, consuming the starting material and forming a number of new hydridic resonances in proton nuclear magnetic resonance (^1^H NMR) spectra <0 ppm. Despite repeated attempts, variation of the reaction conditions did not allow for the clean isolation of these intermediate-hydride species. However, allowing the reaction to proceed for 7 days with 5 equiv of LiAlH_4_ led to the eventual formation of a single product observed spectroscopically, Pn*_2_Ti_2_(μ_2_-H)_5_Li.thf_*x*_ (Scheme 1). Bulk synthesis was achieved by the reaction of (Pn*TiCl)_2_(μ-Cl)_2_ and 5 equiv of LiAlH_4_ in thf over 4 to 7 days. Subsequent crystallization from thf afforded yellow crystals of Pn*_2_Ti_2_(μ_2_-H)_5_Li.thf_*x*_ in 57% yield (*x* = 0.9).

The ^1^H NMR spectrum, in C_4_D_8_O, displays 3 Pn*–*Me* resonances of relative integration 12:12:12, indicating the presence of a time-averaged mirror plane parallel to the Pn*–bridgehead bond. The quantity of coordinated C_4_H_8_O in solution can be measured by integration of ^1^H NMR spectra with values of *x* = 0.8–1.1 typically observed. The thf resonances display no significant deviation from their expected chemical shifts, indicating that exchange of the donor ligand is fast on the NMR timescale. Three discrete hydride environments are observed of relative integration 2:2:1 (−1.09, −2.14, and −4.60 ppm), where the second of these resonances is well resolved as a quartet (^2^*J*_HH_ = 11.7 Hz). Confident assignment of these resonances and their coupling pathways was not possible. Selective magnetization experiments were carried out in C_6_D_6_ with a drop of thf (vide infra), irradiating each hydride environment individually to examine any chemical exchange. No magnetization transfer was observed, indicating that the hydride ligands do not readily exchange and that the structure in solution is rather static.

The zirconium halide precursor, Pn*_2_Zr_2_(μ_2_-Cl)_5_Li.thf_*x*_, as observed for the titanium congener, reacts with 5 equiv of LiAlH_4_ in thf over 7 days and forms a single product as judged by NMR spectroscopy. Following an analogous procedure as described for Pn*_2_Ti_2_(μ_2_-H)_5_Li.thf_*x*_, [Pn*_2_Zr_2_(μ_2_-H)_4_(μ_3_-H)Li.thf_*x*_]_2_ could be isolated in 34% yield as a pale yellow powder.

The ^1^H NMR spectrum qualitatively resembles the titanium congener, with 3 Pn*–*Me* resonances observed indicating the presence of a time-averaged mirror plane parallel to the Pn*–bridgehead bond. The hydride resonances are somewhat shielded relative to the titanium analogue, resonating at 1.83, 1.67, and −0.36 ppm of relative integrations 2:2:1 (c.f. −1.09, −2.14, and −4.60 ppm for Pn*_2_Ti_2_(μ_2_-H)_5_Li.thf_*x*_). The implied *C*_2*v*_ symmetry indicates the formation of dimeric species in solution, as observed for Pn*_2_Ti_2_(μ_2_-H)_5_Li.thf_*x*_. Values for *x* were determined by integration of C_4_H_8_O and Pn*–*Me* resonances and were found to vary between 0.5 and 0.0 ppm. Minimal deviation from the expected chemical shift of thf is observed, suggesting facile exchange of the coordinated donor on the NMR spectroscopic timescale.

**Figure 1:**
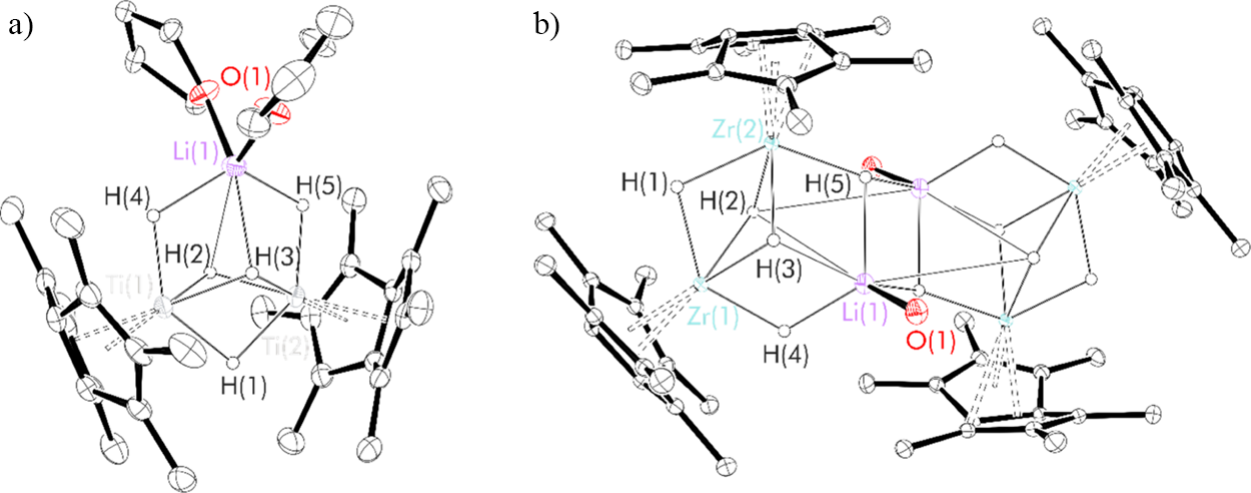
Thermal displacement ellipsoid plots (30% probability) of (a) Pn*_2_Ti_2_(μ_2_-H)_5_Li.thf_2_ and (b) [Pn*_2_Zr_2_(μ_2_-H)_4_(μ_3_-H)Li.thf]_2_. All H atoms apart from the H1–5 have been omitted for clarity.

Selective magnetization experiments indicate facile exchange between the two hydride environments at 1.83 and −0.36 ppm, while magnetization transfer to the third was not observed. This corresponds to exchange between H2/H3 and H4/H5 (Figure 1), while coordination of H1 remains static on the NMR timescale. This observation is slightly unexpected given the stronger metal–ligand bonds expected for zirconium relative to titanium but may result from strain associated with the radially expanded zirconium d-orbitals accommodating a hexagonal bipyramidal geometry.

The observed formation of a stable Ti(IV)-hydride is rather unusual, particularly given its synthesis from LiAlH_4_. Often Ti(IV)-hydrides decompose via loss of H_2_ to give reduced titanium products as is observed for Cp*_2_TiH_2_, which although stable as a solid decomposes in pentane solutions to form Cp*_2_Ti.^12b,17^ Moreover, LiAlH_4_ is generally observed to form reduced titanium-hydride complexes. For example Cp*_2_TiH(Cl) reacts with LiAlH_4_ to give the Ti(III) derivative, Cp*_2_Ti(μ-H_2_)AlH_2_.Et_2_O,^18^ while under more forcing conditions, (Cp^Me4^)_2_TiCl_2_ reacts with LiAlH_4_ to form an allyl-diene complex.^19^ Indeed, while LiAlH_4_ is certainly a viable reducing agent for [Pn*TiCl(μ-Cl)]_2_, it may be that this hexagonal bipyramidal motif offers a degree of kinetic stabilization with respect to H_2_ loss and reduction of the metal centers; Pn* is less sterically demanding than a corresponding bis(Cp) fragment and contributes two fewer electrons. Steric protection by intercalation of LiH and alleviation of electron deficiency by formation of 4 (μ-H) bonds may favor formation of the Ti(IV)–Ti(IV) dimer over any reduced alternative.

The zirconium III/IV redox couple is less accessible than for titanium (first ionization energies: Ti = 4175 kJ/mol; Zr = 3313 kJ/mol),^20^ which leads to the observation of less-facile reduction with LiAlH_4_. Cp*_2_ZrCl_2_ and LiAlH_4_ react to give the Zr(IV) complex, Cp*_2_ZrH(μ-H)_2_AlH_2_, which can be further reacted with ^*n*^BuLi to form the anionic zirconocene hydride, [Cp*_2_ZrH_3_]^−^.^21^ As expected, the three hydride ligands in these complexes are arranged in a coplanar fashion, due to the projection of CpR_2_Zr frontier orbitals in a single plane, in contrast to those observed for [Pn*_2_Zr_2_(μ_2_-H)_4_(μ_3_-H)Li.thf]_2_. While the Cp analogue is less stable,^22^ its synthesis from Cp_2_ZrCl_2_ and LiAlH_4_ can be realized by inclusion of an amine donor to stabilize the complex, forming Cp_2_ZrH(μ-H)_2_AlH_2_(NR_3_) (NR_3_ = quinuclidine, 1-azabicyclo[2.2.2]octane; NMe_3_).^23^

Reduced species can be accessed from Cp_2_ZrCl_2_ by addition of substoichiometric amounts of CoBr_2_ alongside LiAlH_4_, which allows for the formation of [(Cp_2_Zr)_2_(μ-H)](μ-H)_2_AlCl_2_, in which the Zr–H–Al core displays a hexagonal planar geometry similar to that reported for the Pn* complexes.^24^ Hydrogenation of the doubly silylene-bridged *ansa*-zirconocene, [{(Me_2_Si)_2_(η^5^-C_5_H_3_)_2_}ZrMe_2_], forms the structurally characterized trimetallic hydride cluster, [{(Me_2_Si)_2_(η^5^-C_5_H_3_)_2_}Zr]_3_(μ_3_-H)_2_(μ_2_-H)_3_,^25^ although use of the hydride transfer reagent, NaBEt_3_H, produces the more commonly observed zirconium-hydride motif, [{(Me_2_Si)_2_(η^5^-C_5_H_3_)_2_}ZrH]_2_(μ_2_-H)_2_.^26^

### Structure and Bonding of Group 4 Lithium Metal Hydride Clusters: Pn*_2_M_2_(μ_2_-H)_5_Li.thf_*x*_

Single crystals of both Pn*_2_Ti_2_(μ_2_-H)_5_Li.thf_2_ and [Pn*_2_Zr_2_(μ_2_-H)_4_(μ_3_-H)Li.thf]_2_ suitable for X-ray diffraction were grown by slow evaporation of thf solutions at room temperature (Figure 1 and Table 1).

**Table 1:** Comparison of Selected Bond Lengths (Å), Angles (°), and Other Pertinent Structural Metrics

	Pn*_2_Ti_2_(μ_2_-H)_5_Li.thf_2_	[Pn*_2_Zr_2_(μ_2_-H)_4_(μ_3_–H)Li.thf]_2_	Pn*_2_Zr_2_(μ_2_-Cl)_5_Li.thf_2_^33^
fold angle	35.5(1)	32.1(8)	30.0(3)
	34.2(1)	31.2(7)	30.0(3)
M–Pn*_cent_	1.9442(10)	2.0965(7)	2.1095^3^
M–H1 or Cl1	1.83(2)	2.01(2)	2.6431(14)
M–H2/3 or Cl2/3	1.86(3)	2.06(2)	2.6524(13)
M–H4 or Cl4	1.79(3)	1.90(2)	2.5916(12)
M–H5 or Cl5	1.86(3)	2.00(2)	2.5701(13)
H/Cl2–plane_H1/4/5_	1.08(3)	1.28(3)	1.8103(14)
H/Cl3–plane_H1/4/5_	1.10(3)	1.17(3)	1.8378(15)
puckering: Σ|ϕ|^a^	72.80(14)	113.3(16)	143.78(4)
circumference^b^	10.88	11.9	15.16
M–M^c^	2.7874(5)	3.0816(4)	3.6549(9)

a Puckering, given in degrees, is the sum of the magnitude of the dihedral angles around the perimeter of the metal-hydride/chloride core. For a planar structure, this value is 0°.

b The circumference refers to the sum of the bond lengths around the perimeter of the metal-hydride core.

c M–M refers to the Ti–Ti or Zr–Zr distance.

**Figure 2:**
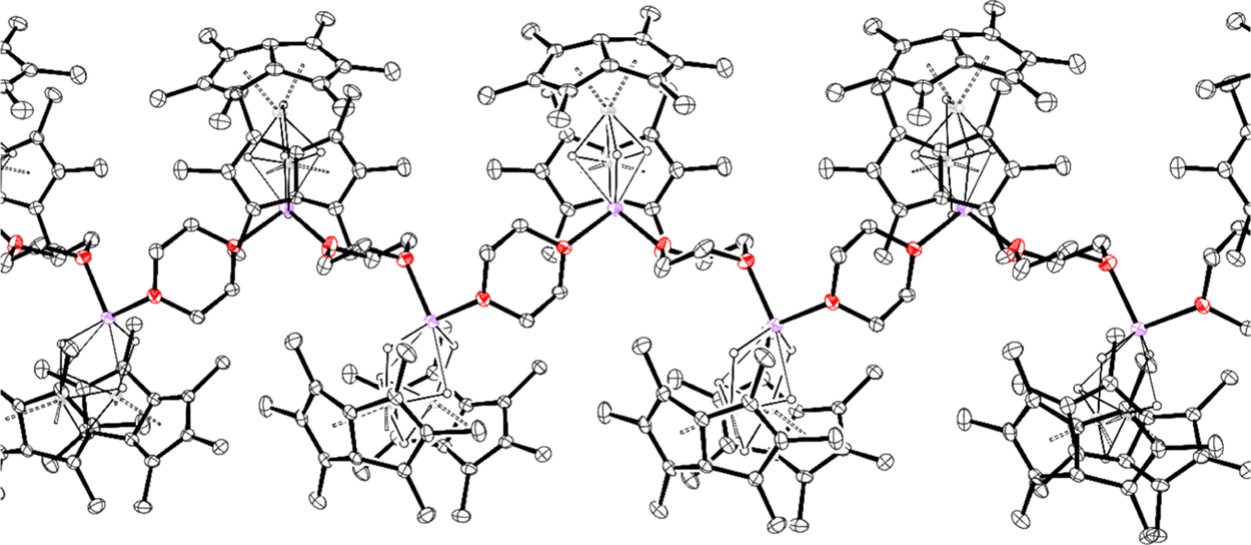
Thermal displacement ellipsoid plot (30% probability) of [Pn*_2_Ti_2_H_5_Li.dioxane]_*n*_. All H atoms apart from H1–5 were omitted for clarity.

The structure is analogous to that of the zirconium(IV) complex, Pn*_2_Zr_2_(μ_2_-Cl)_5_Li.thf_*x*_, and can be similarly rationalized in terms of its Lewis structure as two Ti(IV) centers with intercalated LiH. The central metal-hydride core forms a distorted hexagonal bipyramid, which appears to be a favored structural motif of group 4 Pn* complexes. The exposed lithium ion at the cluster’s apex is weakly bound to the donor solvent, and the complex was also serendipitously crystallized as a mixed thf-benzene solvate. Alternatively, 1,4-dioxane was found to coordinate more strongly, producing similar spectroscopic features to the bis(thf) solvate. Single crystals of the dioxane complex were obtained from slow evaporation of a benzene solution (Figure 2).

The bidentate donor bridges across two neighboring lithium ions, forming a 1D polymer composed of metal-hydride clusters and dioxane linker units. Beyond the polymeric nature of this material, the key bond lengths and angles are similar to those of the bis(thf) complex (see the Supporting Information).

The observation of hexagonal bipyramidal geometry contrasts with the preference of early transition metal Cp_2_M fragments to adopt the more familiar bent metallocene structure found for Cp_2_ZrCl_2_.^27^ Indeed Cp_2_M fragments with vacant coordination sites display a preference for planar coligand bonding, as is observed for Cp_2_TaH_3_,^28,29^ rather than the fourfold coligand coordination that permits formation of this trimetallic bipyramid.

**Figure 3:**
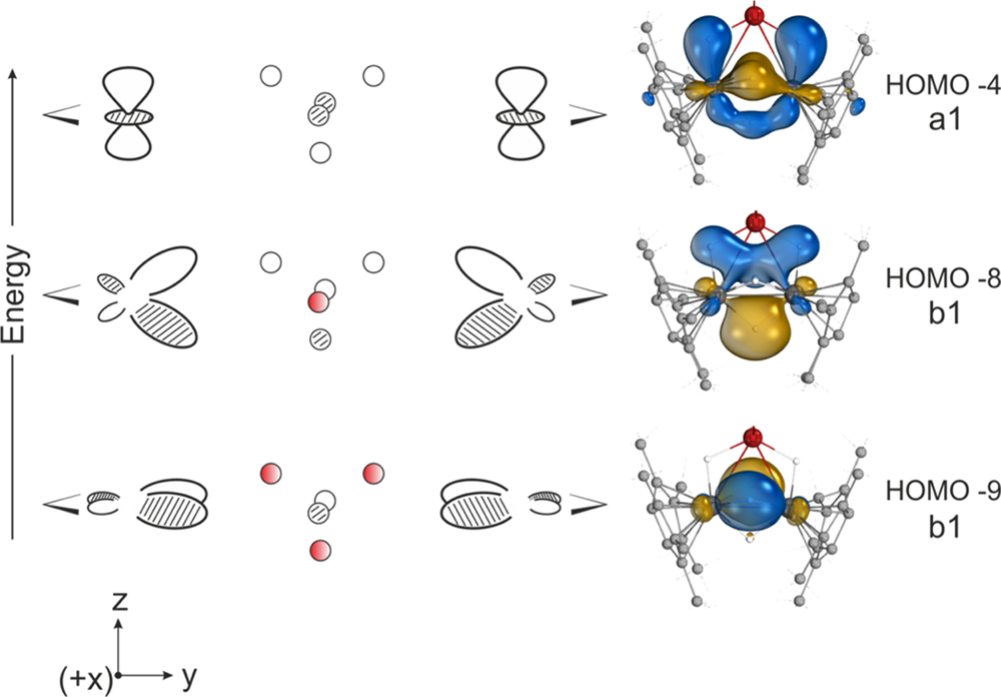
Primary Ti–H bonding orbitals calculated for Pn*_2_Ti_2_(μ_2_-H)_5_Li.thf_2_, illustrating the projection of electron density in two orthogonal planes: *zy* (HOMO-4 and HOMO-8) and *xy* (HOMO-9). Red circles represent hydrogen atoms uninvolved in bonding for each MO.

Density functional theory (DFT) calculations were carried out on Pn*_2_Ti_2_(μ_2_-H)_5_Li.THF_2_ at the B3LYP level of theory,^30^ with Ahlrichs’ triple-ζ basis set employed for heavy elements and hydride ligands.^31^ Geometry optimizations reproduced the experimental data well, although some variation was observed in the orientation of coordinated thf. In the following discussion, the molecular symmetry is approximated as *C*_2*v*_, which is valid in the absence of coordinated THF and assumes that this coordinated solvent has negligible effect on the frontier molecular orbitals. The hexagonal plane is primarily supported by two orbitals of the Pn*Ti fragment: one of a1 symmetry with d_*x*_2_–*y*_2 and d_*z*_2 character, projecting electron density above and below the plane bisecting the Pn*–bridgehead bond, bonding to the whole hydride system, and a second of b1 symmetry, predominantly d_*yz*_ in character. Unlike analogous Cp_2_Ti fragments, an orthogonal b1 symmetric orbital is also accessible, which supports Ti–H2/H3 bonding, securing the hydride caps of the hexagonal bipyramid (Figure 3).

This geometry, rarely encountered for Cp_2_Ti, is therefore a consequence of electron density projected in two orthogonal planes. Interestingly, the calculations also predict a metal–metal bonding interaction between the titanium centers, with a calculated Mayer bond order of 0.616.^32^ Indeed, the distance between the two titanium ions of 2.79 Å (2.82 Å calculated) is well below the sum of the van der Waals radii (4.30 Å for titanium). No molecular orbitals were found to uniquely describe a metal–metal bonding interaction, with this orbital overlap clearly mediated by bridging hydride ligands.

The solid-state structure of [Pn*_2_Zr_2_(μ_2_-H)_4_(μ_3_-H)Li.thf]_2_ is *C*_1_ symmetric, bridging through two apical Li–H fragments, each stabilized by a single thf molecule. The basic structure of the metal-hydride core consists of edge-fused distorted hexagonal bipyramids, similar to those found in Pn*_2_Ti_2_(μ_2_-H)_5_Li.thf_2_, albeit with more extensive puckering observed, which may be a consequence of dimerization. A comparison of key bond lengths and angles is provided in Table 1 for Pn*_2_Ti_2_(μ_2_-H)_4_(μ_3_-H)Li.thf_2_ and [Pn*_2_Zr_2_(μ_2_-H)_4_(μ_3_-H)Li.thf]_2_, along with Pn*_2_Zr_2_(μ_2_-Cl)_5_Li.thf_2_, which also displays this hexagonal bipyramid motif.

Contraction of ionic radii from zirconium to titanium leads to a large increase in the fold angle measured for Pn*_2_Ti_2_(μ_2_-H)_5_Li.thf_2_, along with a decrease in M–Pn*_cent_ and M–H bond lengths. This leads to compression of the hexagonal bipyramid, which can be straightforwardly visualized by considering both the decreased circumference of the metal-hydride core and the decreased H2/3–Plane_H1/4/5_ bond lengths corresponding to narrowing of the pyramidal apices. As would be expected based on a qualitative examination of the structure, more extensive puckering is observed for [Pn*_2_Zr_2_(μ_2_-H)_4_(μ_3_-H)Li.thf]_2_, although given the further increase observed for Pn*_2_Zr_2_(μ_2_-Cl_5_)Li.thf_2_, this is probably not simply a consequence of the former’s greater nuclearity.

DFT calculations were carried at the B3LYP level theory using the same basis set as for Pn*_2_Ti_2_(μ_2_-H)_5_Li.thf_2_, which reproduced the experimental data adequately. Similar metal–metal bonding interactions are predicted with Zr–Zr bond orders of 0.463 and 0.468, lower than those computed for the titanium analogue. This mirrors an increase in the intermetallic distance to 3.12 Å (3.08 Å observed experimentally vs 2.79 Å for Pn*_2_Ti_2_(μ_2_-H)_5_Li.thf_2_), which remains well below the sum of the van der Waals radii for zirconium, 4.60 Å. As before no molecular orbital was calculated to uniquely describe this bonding interaction, with the lower symmetry resulting from dimerization leading to greater orbital mixing.

### Reactivity of Group 4 Lithium Metal Hydride Clusters: Pn*_2_M_2_(μ_2_-H)_5_Li.thf_*x*_

Pn*_2_Ti_2_(μ_2_-H)_5_Li.thf_2_ is surprisingly inert toward many small molecules. When exposed to stoichiometric or excess 2-butyne in C_6_D_6_/THF, no reaction was observed up to 60 °C by ^1^H NMR spectroscopy. Similarly, the complex was found to be inert toward alkenes: *cis*-1,2-diphenylethylene, cyclohexene, and ethylene did not react. Under 1 bar D_2_, trace amounts of HD can be observed by ^1^H NMR spectroscopy (4.51 ppm, t, ^1^*J*_H–D_ = 43 Hz), characteristic of exchange. However, after heating to 60 °C for 4 days, the extent of deuterium incorporation was still insufficient to be resolved by^2^H NMR spectroscopy. The sluggish reactivity of Pn*_2_Ti_2_(μ_2_-H)_5_Li.thf_*x*_ may be a result of the crowded coordination environment around the titanium centers preventing approach of these small molecules.

Although the deuteride complex could not be formed by H–D exchange, its synthesis was achieved by the reaction of LiAlD_4_ in thf over 4–7 days. Following extraction into benzene and subsequent crystallization from thf, Pn*_2_Ti_2_(μ_2_-D)_5_Li.thf_*x*_ was obtained as a crystalline yellow solid in 64% yield. The ^1^H NMR spectrum displays the same Pn*–*Me* resonances unshifted relative to the hydride analogue, with no peaks corresponding to the complex observed below 2.02 ppm. *x* was found to be 0.96 by integration against Pn*–*Me* resonances, with no significant deviation in the THF chemical shift observed. The deuteride resonances were observed by ^2^H NMR spectroscopy at −1.06, −2.12, and −4.49 ppm (c.f. −1.09, −2.14, and −4.60 ppm for the corresponding hydride resonances), demonstrating the successful synthesis of the isotopically labeled complex. Comparison of FTIR spectra of Pn*_2_Ti_2_(μ_2_-H)_5_Li.thf_*x*_ and Pn*_2_Ti_2_(μ_2_-D)_5_Li.thf_*x*_ allows for the definitive assignment of Ti–H/D stretching frequencies. An intense peak at 1507 cm^–1^ is observed for the hydride complex, which is shifted to 1099 cm^–1^ on perdeuteration, which is in good agreement with the value calculated using the reduced mass formula (1077 cm^–1^). A second hydride-stretch observed at 1291 cm^–1^ is red-shifted to 941 cm^–1^, in close agreement with a predicted value of 922 cm^–1^.

In contrast, for [Pn*_2_Zr_2_(μ_2_-D)_4_(μ_3_-D)Li.thf_*x*_]_2_, there is facile D_2_ incorporation, with complete disappearance of hydride resonances observed after 4 days under 1 bar D_2_ at room temperature. This is accompanied by HD formation, which can be clearly resolved by ^1^H NMR spectroscopy. The deuteride complex was synthesized on a preparative scale by the reaction of Pn*_2_Zr_2_Cl_5_Li.THF_*x*_ and LiAlD_4_ in thf. Extraction into benzene and crystallization from thf allowed for the isolation of [Pn*_2_Zr_2_(μ_2_-D)_4_(μ_3_-D)Li.thf_*x*_]_2_ in 41% yield as a yellow powder.

### A Group 4 Trimetallic Hydride Cluster: Pn*_3_Ti_3_(μ_2_-H)_3_(μ_3_-H)

**Figure 4:**
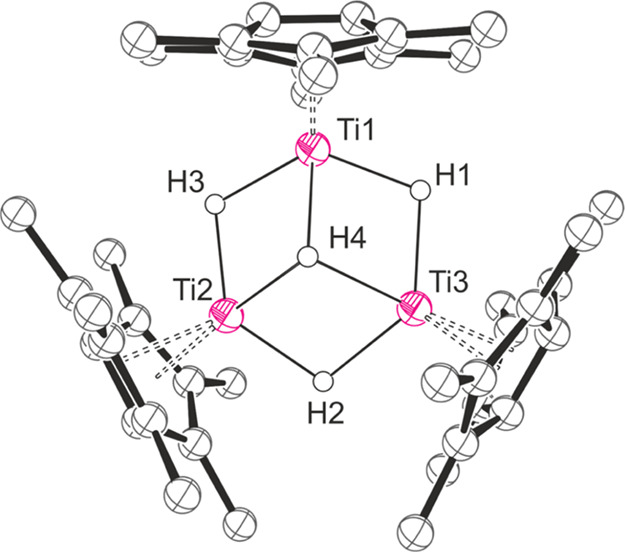
Thermal displacement ellipsoid plot (30% probability) of Pn*_3_Ti_3_(μ_2_-H)_3_(μ_3_-H). All H atoms apart from H1–4 are omitted for clarity. H4 is shown in one of the two partially occupied positions.

**Scheme 2:**
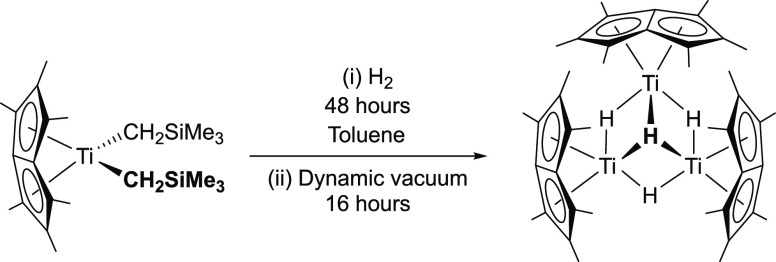
Synthesis of Pn*_3_Ti_3_(μ_2_-H)_3_(μ_3_-H)

The purple titanium alkyl precursor, Pn*Ti(CH_2_SiMe_3_)_2_, was first synthesized by the salt elimination reaction of [Pn*TiCl](μ–Cl)_2_ with 2 equiv of NaCH_2_SiMe_3_. On exposing a C_6_D_6_ solution of Pn*Ti(CH_2_SiMe_3_)_2_ to H_2_, the solution turned black-green over the course of 48 h. ^1^H NMR spectra indicated the consumption of the starting material and the formation of new diamagnetic and paramagnetic species alongside SiMe_4_ (Scheme 2). Only after drying the sample under dynamic vacuum was a clean, diamagnetic complex obtained, with 2 Pn*–*Me* resonances resolved at 2.31 and 2.16 ppm in the ^1^H NMR spectrum, alongside a single hydride resonance at −2.91 ppm integrated to 4H. Single crystals suitable for an X-ray diffraction study were grown from slow evaporation of a benzene solution with 1 drop of added toluene, which confirms the formation of the trimetallic hydride cluster Pn*_3_Ti_3_(μ_2_-H)_3_(μ_3_-H) (Figure 4 and Table 2).

**Table 2:** Comparison of Selected Bond Lengths (Å), Angles (°), and Other Pertinent Structural Metrics of Pn*_3_Ti_3_(μ_2_-H)_3_(μ_3_-H) with [{(Me_2_Si)_2_(η^5^-C_5_H_3_)_2_}Zr]_3_H_5_

	Pn*_3_Ti_3_(μ_2_-H)_3_(μ_3_-H)	[{(Me_2_Si)_2_(η^5^-C_5_H_3_)_2_}Zr]_3_H_5_^25^
fold angle	33.43(10)	-
	32.27(9)	
	32.27(9)	
M–Pn*_cent_/Cp_cent_	1.9810(10)	2.2648(10)
M–H1	1.78(3)	1.963(19)
M–H2	1.84(3)	2.16(2)
M–H3	1.84(3)	1.94(2)
M–H4	1.995(16)	1.94(2)
M–H5		2.16(2)
H4–plane_H1/3/4_	1.17(2)	0.93(2)
H5–plane_H1/3/4_		0.93(2)
puckering: Σ|ϕ|^a^	82(6)	28.4(11)
circumference^b^	10.91(24)	11.67(12)
M–M^c^	2.9454(9)	3.2939(10)

a Puckering, given in degrees, is the sum of the magnitude of the dihedral angles around the perimeter of the metal-hydride core. For a planar structure, this value is 0°.

b The circumference refers to the sum of the bond lengths around the perimeter of the metal-hydride core.

c M–M given as an average of three values.

### Structure and Bonding of a Group 4 Trimetallic Hydride Cluster: Pn*_3_Ti_3_(μ_2_-H)_3_(μ_3_-H)

The central metal-hydride core adopts a hexagonal pyramidal geometry with one face uncapped, similar to the complexes discussed previously. The hydride atoms were crystallographically located, although H4 was found to be partially disordered across both faces of the hexagon. A comparison of key bond lengths and angles is provided in Table 2 alongside [{(Me_2_Si)_2_(η^5^-C_5_H_3_)_2_}Zr]_3_H_5_,^25^ which is the closest structural analogue found in the literature.

The fold angles are shallower for Pn*_3_Ti_3_(μ_2_-H)_3_(μ_3_-H) than for Pn*_2_Ti_2_(μ_2_-H)_5_Li.thf_2_, which is likely a result of formal reduction of two titanium centers to Ti(III). Elongation of the apical hydride–titanium bond is observed, which is also mirrored in [{(Me_2_Si)_2_(η^5^-C_5_H_3_)_2_}Zr]_3_H_5_. Slightly surprising is the contraction of bipyramid apices for [{(Me_2_Si)_2_(η^5^-C_5_H_3_)_2_}Zr]_3_H_5_ relative to Pn*_3_Ti_3_(μ_2_-H)_3_(μ_3_-H), despite an increased ionic radius in the former case. The titanium complex is more puckered than the zirconium analogue, which may be facilitated by the symmetrical displacement of capping hydride ligands in [{(Me_2_Si)_2_(η^5^-C_5_H_3_)_2_}Zr]_3_H_5_. Similarly, Pn*_2_Ti_2_(μ_2_-H)_5_Li.thf_2_, with two capping hydride ligands, displays a less-puckered structure than Pn*_3_Ti_3_(μ_2_-H)_3_(μ_3_-H), suggesting that the structural stabilization afforded by this symmetrical ligand placement outweighs any distorting effects resulting from hetero-metal incorporation. Despite this, the circumference of Pn*_3_Ti_3_(μ_2_-H)_3_(μ_3_-H) is almost identical to that found for Pn*_2_Ti_2_(μ_2_-H)_5_Li.thf_2_, although a slight increase in average Ti–Ti distance is observed.

Pn*_3_Ti_3_(μ_2_-H)_3_(μ_3_-H) is a mixed-valence hydride cluster with d-electron count d^0^–d^1^–d^1^, which might be expected to behave as a paramagnet. However, spectroscopic evidence indicates that it is diamagnetic. DFT calculations were therefore carried out to probe the origin of this diamagnetism, gain insight into the nature of bonding in the complex, and investigate energetically accessible derivatives that may aid in the rationalization of the electron paramagnetic resonance (EPR) data.

**Figure 5:**
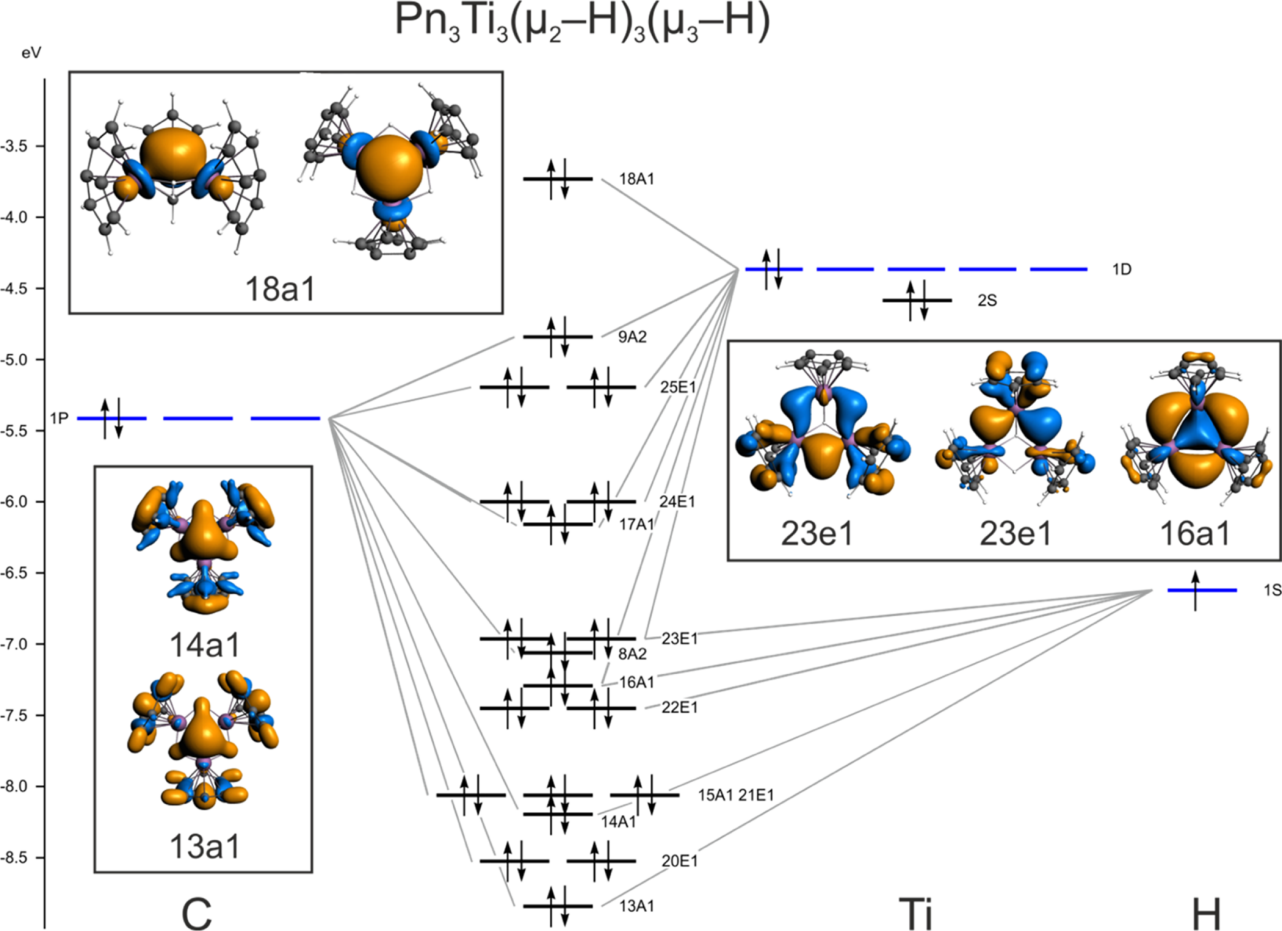
Fragment molecular orbital diagram for Ti_3_Pn*_3_(μ_2_-H)_3_(μ_3_-H), with key Ti–Ti and Ti–H bonding interactions highlighted.

Density functional calculations were carried out using the Amsterdam density functional package^34^ and triple-ζ quality basis sets augmented with a one polarization function (ADF basis TZP). The local density approximation of Vosko, Wilk, and Nusair^35^ was used together with the exchange correlation corrections of Becke and Perdew (BP86).^36^ The geometries of both Ti_3_Pn_3_(μ_2_-H)_3_(μ_3_-H) and Ti_3_Pn*_3_(μ_2_-H)_3_(μ_3_-H) were successfully optimized with *C*_3*v*_ symmetry found for the Pn complex and the Ti_3_H_4_ core of the Pn* analogue. For simplicity, a bonding analysis was carried out on Ti_3_Pn_3_(μ_2_-H)_3_(μ_3_-H) (Figure 5).

The HOMO, 18a1, is a cluster-based bonding orbital composed primarily of metal d_*z*_2 orbitals, projecting onto the vacant face of the hexagonal pyramid. The diamagnetism of the complex can be understood to result from the overlap of these d_*z*_2 orbitals, which accommodates the two otherwise unpaired electrons. The next six lower energy orbitals describe titanium–pentalene bonding interactions, which are followed by a set of orbitals, some of which have Ti–H bonding characteristics (23e1 and 16a1) that describe the Ti–H–Ti 3c-2e bonds in a localized bonding model. The apical hydride bonding interaction is primarily exhibited lower in the manifold in orbitals 14a1 and 13a1, with the edge-bridging hydrides contributing to lesser extent.

**Figure 6:**
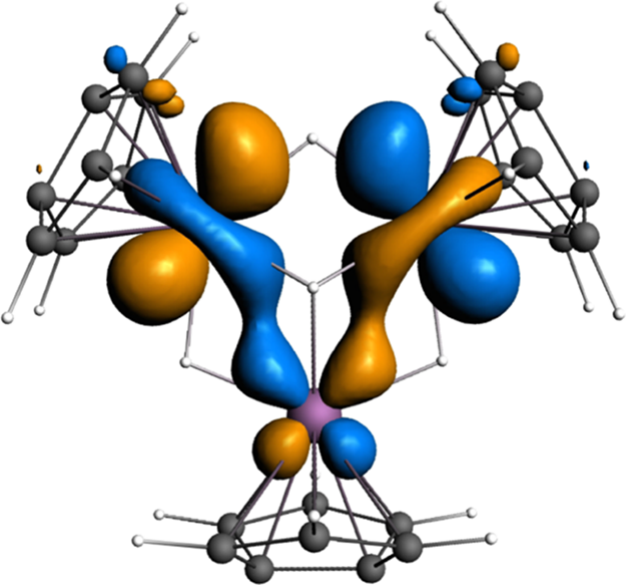
SOMO calculated for the paramagnetic hydride complex, Ti_3_Pn_3_(μ_2_–H)_3_(μ_3_–H)_2_.

Calculations were also performed on Ti_3_Pn*_3_(μ_2_-H)_3_(μ_3_-H)_2_, paramagnetic species, and Ti_3_Pn_3_(μ_2_-H)_3_(μ_3_-H)_2_. Various starting structures were attempted, the lowest energy of which was a *D*_3*h*_ symmetric structure with the additional hydride atom localized on the exposed face of the hexagonal pyramid. The extra electron occupies a SOMO, 26e1, localized on the three titanium atoms (Pn analogue shown in Figure 6). Jahn–Teller distortion from pure *D*_3*h*_ symmetry is therefore predicted, but given the weak bonding/antibonding characteristics of the 26e1 orbital, such a distortion might not be static. As discussed in relation to Pn*_2_Ti_2_(μ_2_-H)_5_Li.thf_2_, this hexagonal bipyramidal metal-hydride core can be understood to result from the orthogonal disposition of the PnTi frontier molecular orbitals. The most closely related complex previously reported is the trimetallic zirconium hydride cluster [{(Me_2_Si)_2_(η^5^-C_5_H_3_)_2_}Zr]_3_(μ_3_-H)_2_(μ_2_-H)_3_, formally Zr(IV)/Zr(IV)/Zr(III), which was identified in the solid-state and also contains the same structural motif as for Ti_3_Pn*_3_(μ_2_-H)_3_(μ_3_-H)_2._^25^

### Reactivity of a Group 4 Trimetallic Hydride Cluster: Pn*_3_Ti_3_(μ_2_-H)_3_(μ_3_-H)

A solution of Pn*_3_Ti_3_(μ_2_-H)_3_(μ_3_-H) in C_7_D_8_ was exposed to 1 bar D_2_ at 298 K and analyzed by NMR spectroscopy after 15 minutes. The ^1^H NMR spectrum indicated the presence of paramagnetic products even after this short reaction time, with broad overlapping resonances observed between 5 and 1 ppm, alongside the starting material and other unidentified diamagnetic resonances. ^2^H NMR spectra displayed a new resonance at −3.1 ppm, resulting from deuterium exchange, although no resonance corresponding to HD could be observed. After 48 h, the ^1^H NMR spectrum was somewhat similar to that observed under H_2_. However, clear differences can be observed both in the number and position of the paramagnetic and diamagnetic components (Supporting Information).

The rapid reaction with D_2_ contrasts dramatically with the slow exchange observed for Pn*_2_Ti_2_(μ_2_-H)_5_Li.thf_*x*_, which is presumably a consequence of the vacant coordination site presented by Pn*_3_Ti_3_(μ_2_-H)_3_(μ_3_-H). After exposing the paramagnetic mixture to dynamic vacuum and redissolving in C_7_D_8_, the diamagnetic deuterated complex, Pn*_3_Ti_3_(μ_2_-D)_3_(μ_3_-D), could be observed by NMR spectroscopy. Two Pn*–*Me* resonances at 2.30 and 2.15 ppm are the only ones observed by ^1^H NMR spectroscopy, with a corresponding deuteride resonance at −3.1 ppm. Pn*_3_Ti_3_(μ_2_-D)_3_(μ_3_-D) was synthesized on a preparative scale by an analogous method to Pn*_3_Ti_3_(μ_2_-H)_3_(μ_3_-H), with D_2_ gas instead of H_2_. The product was obtained in 65% yield as a black crystalline solid.

FTIR spectroscopy of the hydride and deuteride complexes displays stretches at 1310 and 936 cm^–1^, respectively, which is in close agreement with values predicted by the reduced mass formula (1310 and 944 cm^–1^). A new peak at 1043 cm^–1^ in the spectrum of Pn*Ti_3_D_4_ could not be related by its reduced mass to a corresponding peak in the spectrum of Pn*_3_Ti_3_(μ_2_-H)_3_(μ_3_-H) (expected value 1457 cm^–1^) due to overlap with an intense broad peak at 1444 cm^–1^ assigned to Pn* C=C bond stretching.

Given the observation of paramagnetism under H_2_/D_2_, Pn*_3_Ti_3_(μ_2_-H)_3_(μ_3_-H) was studied by X-band EPR spectroscopy in the presence of these gases. A toluene solution of Pn*_3_Ti_3_(μ_2_-H)_3_(μ_3_-H) was pressurized with 1 bar H_2_ in a J-Youngs Quartz EPR tube, and spectra were collected after 30 min, 6 h, and 48 h.

Complicated speciation was inferred from the observation of a number of paramagnetic components between *g* = 1.96 and 1.98. Due to the overlap and weak intensity of these resonances, it is not possible to confidently assign the number of minor components. The major component at *g* = 1.993 is clearly resolved 30 minutes after gas addition and is observed to increase in relative intensity over the course of 48 h, with intermediate conversion observed at 6 h. That the additional components are the result of speciation and not decomposition was confirmed by repeating the reaction at a fivefold increase in concentration (25 mM vs 5 mM), which produced these minor components to the same extent. Indeed, the presence of both paramagnetic and diamagnetic components by ^1^H NMR spectroscopy under H_2_ pressure further alludes to the formation of multiple products and it could be envisaged that variation in nuclearity and hydride/deuteride content under H_2_/D_2_ may lead to the observed speciation. Unfortunately, no hyperfine coupling to the hydride ligands was observed, which limits additional insight into the nature of these complexes.

Repeating the reaction with D_2_, similar reactivity was observed. The same resonance at *g* = 1.993 is observed, formed within 30 minutes and increasing in intensity over the course of 48 h. However, the distribution of minor components is altered relative to H_2_, with a more intense resonance at *g* = 1.972 resolved under D_2_ after 48 h. No additional splitting of peaks is observed (*D*, *S* = 1; *H*, *S* = 1/2), confirming the absence of hyperfine coupling at the experimental resolution.

Attempts to crystallize the major product from saturated toluene solutions of Pn*_3_Ti_3_(μ_2_-H)_3_(μ_3_-H) under H_2_ pressure were unsuccessful, preventing the conclusive identification of these paramagnetic complexes in the solid state. Some plausible structures for this major paramagnetic component could therefore be proposed. Pn*_3_Ti_3_(μ_2_-H)_3_(μ_3_-H)_2_, related to the diamagnetic starting complex by incorporation of an additional hydride ligand across the open face of the hexagonal bipyramid. Alternatively, it could be envisaged that variation in the nuclearity of the metal-hydride product could occur, forming complexes such as the mixed valence hydride dimer, (Pn*Ti)_2_(μ-H)_3_, displaying a Ti/H ratio of 1:1.5 (1:1.33 for the diamagnetic starting complex).

An interesting comparison can be drawn between Pn*_3_Ti_3_(μ_2_-H)_3_(μ_3_-H) and the titanium half-sandwich hydride, [(Cp′Ti)_3_(μ_3_-H)(μ_2_-H)_6_] (Cp′ = C_5_Me_4_SiMe_3_), formed by hydrogenation of Cp′Ti(CH_2_SiMe_3_)_3_.^10c^ The structure can be considered analogous to that of Pn*_3_Ti_3_(μ_2_-H)_3_(μ_3_-H), only with six hydride ligands bracing the hexagonal plane to account for the substitution of Pn* for a monoanionic carbocycle, Cp′. This contrasts with the tetrametallic clusters observed following hydrogenation of Cp′M(CH_2_SiMe_3_)_3_ (M = Zr, Hf), where all four metal centers are in the +3 oxidation state.^10d^

[(Cp′Ti)_3_(μ_3_-H)(μ_2_-H)_6_] displays remarkable reactivity, cleaving the dinitrogen triple bond to form two bridging [NH]^2–^ ligands in 90% yield,^10c^ as well as activating benzene to form methylcyclopentenyl derivatives.^10a,10b^ Unfortunately, despite their structural similarity, comparable transformations were not observed with in Pn*_3_Ti_3_(μ_2_-H)_3_(μ_3_-H). The complex is stable in benzene and does not react with N_2_ (up to 4 bar). With CO, decomposition is observed, similar to the reported reactivity with Pn*_2_Ti_2_(μ_2_-H)_5_Li.thf_*x*_, while with CO_2_ no evidence for hydride transfer is observed although a number of new hydride resonances could be resolved by NMR spectroscopy, perhaps resulting from the formation of titanium–oxide–hydride derivatives. The complex is unreactive toward 2-butyne, although reactivity is observed with ethylene. Following the reaction by ^1^H NMR spectroscopy, slow consumption of the starting material was observed alongside the formation of a large number of Pn*–*Me* and the appearance of eight new hydride environments in the region −0.49 to −6.54 ppm. This divergent reactivity of complexes that appear electronically and structurally so related highlights the importance of the ancillary ligand set in directing the chemistry of metal hydrides. Although clean reaction with small molecules could not be realized, it is interesting to contrast the reactivity of Pn*_3_Ti_3_(μ_2_-H)_3_(μ_3_-H) to the stability of Pn*_2_Ti_2_(μ_2_-H)_5_Li.thf_*x*_ under similar conditions, which may be driven by the presence of a suitable vacancy in the coordination sphere of the metal centers in Pn*_3_Ti_3_(μ_2_-H)_3_(μ_3_-H).

## Conclusions

The preparation of new permethylpentalene, Pn*, metal-hydride complexes has been described, Pn*_2_Ti_2_(μ_2_-H)_5_Li.thf_*x*_ and [Pn*_2_Zr_2_(μ_2_-H)_5_Li.thf_*x*_]_2_, which can both be accessed from the corresponding M(IV) chloride precursor and LiAlH_4_ in thf. A number of intermediate hydride species were observed spectroscopically. The titanium-hydride complex is not fluxional on the NMR spectroscopic timescale and does not readily exchange with D_2_, while the zirconium congener undergoes chemical exchange between two of the three observed hydride environments. As expected, given its fluxional solution-phase behavior, the zirconium complex also readily exchanged with D_2_ gas in a C_6_D_6_/thf solution. The deuteride complexes, Pn*_2_Ti_2_(μ_2_-D)_5_Li.thf_*x*_ and [Pn*_2_Zr_2_(μ_2_-D)_5_Li.thf_*x*_]_2_, were successfully synthesized using LiAlD_4_ and characterized by NMR and FTIR spectroscopy. Both complexes were structurally characterized with a variety of solvents coordinated to the apical lithium atom and demonstrate an unusual hexagonal bipyramidal geometry. In the case of [Pn*_2_Zr_2_(μ_2_-H)_4_(μ_3_-H)Li.thf]_2_, edge-fused bipyramids are observed in the solid state, forming a more puckered hydride cluster. This geometry has only rarely been encountered previously and can be rationalized by DFT calculations as the resulting frontier orbital electron density projected in two orthogonal planes, in contrast with the coplanar orientation found for Cp_2_M complexes.

The diamagnetic mixed-valence titanium hydride cluster, in Pn*_3_Ti_3_(μ_2_-H)_3_(μ_3_-H), was also investigated. Surprisingly, under H_2_, the complex reversibly forms a large number of unidentified paramagnetic components, which are all converted to Pn*_3_Ti_3_(μ_2_-H)_3_(μ_3_-H) under dynamic vacuum overnight. The proposed structure was crystallographically confirmed and further corroborated by DFT calculations, which illustrated the formation of a metal-cluster bonding orbital responsible for the diamagnetism of the complex. These new hydride complexes provide interesting comparisons, both in terms of solid-state structures and reactivity, to related cyclopentadienyl analogues.

## Experimental Details

All manipulations were carried out using standard Schlenk line or drybox techniques under an atmosphere of dinitrogen or argon. Protio solvents were degassed by sparging with dinitrogen, dried by passing through a column of activated sieves (pentane, hexane, toluene, and benzene) and stored over potassium mirrors, or distilled from sodium metal (thf) and stored over activated 4 Å molecular sieves, or distilled from sodium-potassium alloy (diethyl ether) and stored over a potassium mirror. Deuterated solvents were dried over potassium (C_6_D_6_, C_7_D_8_) or CaH_2_ (C_4_D_8_O), distilled under reduced pressure, and freeze–pump–thaw degassed three times prior to use.

^1^H NMR spectra were recorded at 298 K, unless otherwise stated, on Bruker AVIII 400 nanobay or Bruker AVIII 500 spectrometers, and ^13^C{^1^H} or ^13^C NMR spectra, on the same spectrometers at operating frequencies of 100 and 125 MHz, respectively. Two-dimensional ^1^H**–**^1^H and ^13^C**–**^1^H correlation experiments were used, when necessary, to confirm ^1^H and ^13^C NMR assignments. All NMR spectra were referenced internally to residual protio solvent (^1^H) or solvent (^13^C) resonances and are reported relative to tetramethylsilane (δ = 0 ppm). Chemical shifts are quoted in δ (ppm), and coupling constants, in Hertz. Elemental analyzes were carried out at London Metropolitan University. FTIR spectra were prepared in a glove box as pressed KBr discs. Spectra were recorded on a Nicolet iS5 Thermo Scientific spectrometer. Pn*_3_Ti_3_(μ_2_-H)_3_(μ_3_-H) was studied by X-band EPR spectroscopy in the presence of H_2_ or D_2_. A toluene solution of Pn*_3_Ti_3_(μ_2_– H)_3_(μ_3_–H) was pressurized with 1 bar H_2_ in a J-Young Quartz EPR tube, and spectra were collected after 30 minutes, 6 h, and 48 h.

The following compounds were synthesized according to a literature procedure: [Pn*TiCl(μ-Cl)]_2_ and [ZrPn*(μ-Cl)_3/2_]_2_(μ-Cl)_2_Li.thf_*x*_.^33^

### Pn*_2_Ti_2_(μ_2_-H)_5_Li.thf_*x*_

[Pn*TiCl](μ–Cl)_2_ (0.250 g, 0.410 mmol) and LiAlH_4_ (0.777 g, 2.05 mmol) were dissolved in thf (10 mL) and stirred at room temperature for 4 days. An aliquot was taken, and if necessary, further LiAlH_4_ was added and stirred for 36 h. Once completed, the volatiles were removed under vacuum and the gray solid was extracted with benzene (4 × 7 mL) and filtered through celite. The combined extracts were concentrated to *ca.* 10 mL, and the mixture was lyophilized under dynamic vacuum to give a flocculant brown solid. This was dissolved in a minimum volume of thf (3 × 3 mL) and filtered a second time through celite. This solution was concentrated to *ca.* 2–3 mL and cooled to −80 °C for 1 week, depositing bright yellow crystals of Pn*_2_Ti_2_(μ_2_-H)_5_Li.thf_2_, which were isolated by filtration and washed once with −78 °C pentane (1 × 3 mL). The solid was dried under dynamic vacuum for 4 h, allowing for the isolation of Pn*_2_Ti_2_(μ_2_-H)_5_Li.thf_*x*_ in 57% yield (0.127 g, 0.234 mmol, *x* = 0.9, determined by ^1^H NMR spectroscopy). Single crystals of Pn*_2_Ti_2_(μ_2_-H)_5_Li.thf_2_ suitable for an X-ray diffraction study were grown by slow evaporation of a thf solution. ^1^H NMR (400 MHz, 298 K, C_4_D_8_O) δ (ppm): 2.34 (s, 6H, 1,7-*Me*–Pn*), 2.28 (s, 6H, 3,5-*Me*–Pn*), 1.96 (s, 6H, 2,6-*Me*-Pn*), −1.09 (br s, ν_1/2_ = 29 Hz, 2H, Ti–*H*), −2.14 (q, 2H, ^2^*J*_H–H_ = 11.7 Hz, Ti–*H*), −4.60 (m, 1H, Ti–*H*). ^1^H NMR (400 MHz, 298 K, C_6_D_6_/C_4_D_8_O 9:1) δ (ppm): 2.57 (s, 6H, 1,7-*Me*–Pn*), 2.52 (s, 6H, 3,5-*Me*–Pn*), 2.08 (s, 6H, 2,6-*Me*–Pn*), −0.94 (m, 2H, Ti–*H*), −1.94 (m, 2H, Ti–*H*), −4.36 (m, 1H, Ti–*H*). ^13^C{^1^H} NMR (100 MHz, 298 K, C_4_D_8_O) δ (ppm): 122.2 (4-Pn*), 121.6 (2,6-Pn*), 119.9 (8-Pn*), 109.7 (1,7-Pn*), 108.6 (3,5-Pn*), 14.7 (3,5-*Me*–Pn*), 14.4 (1,7-*Me*–Pn*), 10.9 (2,6-*Me*–Pn*). ^7^Li NMR (156 MHz, 298 K, C_4_D_8_O) δ (ppm): 1.22. FTIR (KBr disc, cm^–1^): 2977 (s), 2952 (s), 2885 (br, s), 1507 (br, s), 1454 (br, s), 1291 (br, s), 1052 (m), 1024 (m), 896 (w). Anal calcd (found) for C_31.6_H_48.2_LiTi_2_O: C, 69.62 (68.90); H, 8.85 (8.80) – based on *x* = 0.9.

### Pn*_2_Ti_2_(μ_2_-D)_5_Li.thf_*x*_

Synthesized using an analogous route to Pn*_2_Ti_2_(μ_2_-H)_5_Li.thf_*x*_ with LiAlD_4_ ([Pn*TiCl](μ-Cl)_2_ (0.250 g, 0.410 mmol), LiAlD_4_ (0.861 g, 2.05 mmol)). Pn*_2_Ti_2_(μ_2_-D)_5_Li.thf_*x*_ was isolated in 64% yield as a yellow crystalline solid (0.146 g, 0.262 mmol, *x* was determined to be 0.96 by ^1^H NMR spectroscopy). ^1^H NMR (400 MHz, 298 K, C_6_D_6_/C_4_D_8_O 9:1) δ (ppm): 2.51 (s, 6H, 1,7-*Me*–Pn*), 2.46 (s, 6H, 3,5-*Me*–Pn*), 2.02 (s, 6H, 2,6-*Me*-Pn*). ^13^C{^1^H} NMR (100 MHz, 298 K, C_6_D_6_:C_4_D_8_O 9:1) δ (ppm): 122.8 (4-Pn*), 122.2 (2,6-Pn*), 119.4 (8-Pn*), 110.1 (1,7-Pn*), 108.1 (3,5-Pn*), 14.2 (3,5-*Me*–Pn*), 13.7 (1,7-*Me*–Pn*), 10.4 (2,6-*Me*–Pn*). ^2^H NMR (76.7 MHz, 298 K, C_6_D_6_/C_4_D_8_O 9:1) δ (ppm): −1.06 (2D, Ti–*D*), −2.12 (2D, Ti–*D*), −4.49 (1D, Ti–*D*). ^7^Li NMR (156 MHz, 298 K, C_6_D_6_/C_4_D_8_O 9:1) δ (ppm): 1.39. FTIR (KBr disc, cm^–1^): 2977 (s), 2952 (s), 2887 (br, s), 1452 (br, m), 1377 (m), 1099 (br, m), 1052 (s), 941 (s), 805 (w). Anal calcd (found) for C_32_H_44_D_5_LiTi_2_O: C, 68.95 (67.83); H, 9.76 (9.42).

### [Pn*_2_Zr_2_(μ_2_-H)_4_(μ_3_-H)Li.thf_*x*_]_2_

[ZrPn*(μ–Cl)_3/2_]_2_(μ–Cl)_2_Li.thf_2_ (0.200 g, 0.226 mmol) and LiAlH_4_ (0.430 g, 1.13 mmol) were dissolved in thf (10 mL) and stirred at room temperature for 4 days. An aliquot was taken, and if necessary, further LiAlH_4_ was added and stirred for 36 h. Once complete, the volatiles were removed under vacuum and the gray solid was extracted with benzene (4 × 7 mL) and filtered through celite. The combined extracts were concentrated to *ca.* 10 mL, and the mixture was lyophilized under dynamic vacuum to give a flocculant pale yellow solid. This was redissolved in a minimum volume of thf (3 × 4 mL) and filtered a second time through celite. The volatiles were removed under vacuum, slurried in benzene (5 mL), and lyophilized under dynamic vacuum, yielding [Pn*_2_Zr_2_(μ_2_-H)_4_(μ_3_-H)Li.thf_*x*_]_2_ in 34% yield (46 mg, 0.038 mmol, *x* = 0.5, determined by ^1^H NMR spectroscopy). Single crystals of [Pn*_2_Zr_2_(μ_2_-H)_4_(μ_3_-H)Li.thf_2_]_2_ suitable for an X-ray diffraction study were grown by slow evaporation of a thf solution. ^1^H NMR (400 MHz, 298 K, C_4_D_8_O) δ (ppm): 2.30 (s, 6H, 1,7-*Me*–Pn*), 2.28 (s, 6H, 3,5-*Me*–Pn*), 2.03 (s, 6H, 2,6-*Me*–Pn*), 1.67 (s, 2H, Zr–*H*), 1.51 (s, 2H Zr–*H*), −0.55 (s, 1H, Zr–*H*). ^1^H NMR (400 MHz, 298 K, C_6_D_6_/C_4_D_8_O 8:2) δ (ppm): 2.54 (s, 6H, 1,7-*Me*–Pn*), 2.51 (s, 6H, 3,5-*Me*–Pn*), 2.14 (s, 6H, 2,6-*Me*–Pn*), 1.89 (s, 2H, Zr–*H*), 1.73 (s, 2H Zr–*H*), −0.31 (s, 1H, Zr–*H*). ^13^C{^1^H} NMR (100 MHz, 298 K, C_4_D_8_O) δ (ppm): 122.0120.0 (4,8-Pn* could not be distinguished by heteronuclear multiple bond correlation (HMBC)), 125.0, 105.2, 104.7 (1,2,3,5,7-Pn*, could not be distinguished by HMBC), 13.6 (3,5-*Me*-Pn*), 13.3 (1,7-*Me*–Pn*), 10.7 (2,6-*Me*–Pn*). FTIR (KBr disc, cm^–1^): 2976 (s), 2955 (s), 2905 (s), 2863 (s), 1451 (m), 1380 (m), 1260 (s), 1099 (w), 1046 (w), 1028 (w). Anal calcd (found) for C_64_H_98_Li_2_O_2_Zr_4_: C, 60.14 (61.90); H, 7.73 (7.88) – based on *x* = 1.

### [Pn*_2_Zr_2_(μ_2_-D)_4_(μ_3_-D)Li.thf_*x*_]_2_

Synthesized by an analogous route to [Pn*_2_Zr_2_(μ_2_-H)_4_(μ_3_-H)Li.thf_*x*_]_2_ ([ZrPn*(μ-Cl)_3/2_]_2_(μ-Cl)_2_Li.thf_2_ (0.200 g, 0.226 mmol), LiAlD_4_ (0.475 g, 1.13 mmol)). [Pn*_2_Zr_2_(μ_2_-D)_4_(μ_3_-D)Li.thf_*x*_]_2_ was isolated in 41% yield (0.058 g, 0.046 mmol, *x* = 0.8, determined by ^1^H NMR spectroscopy). ^1^H NMR (400 MHz, 298 K, C_6_D_6_:C_4_D_8_O 8:2) δ (ppm): 2.51 (s, 6H, 1,7-*Me*–Pn*), 2.49 (s, 6H, 3,5-*Me*–Pn*), 2.13 (s, 6H, 2,6-*Me*–Pn*). ^13^C{^1^H} NMR (100 MHz, 298 K, C_6_D_6_/C_4_D_8_O 8:2) δ (ppm): 122.7, 120.2 (4,8-Pn* could not be distinguished by HMBC), 125.4, 104.9, 104.1 (1,2,3,5,7-Pn*, could not be distinguished by HMBC), 14.0 (3,5-*Me*–Pn*), 13.7 (1,7-*Me*–Pn*), 11.1 (2,6-*Me*–Pn*). ^2^D NMR (76.7 MHz, 298 K, C_6_D_6_/C_4_D_8_O 8:2) δ (ppm): 1.77 (Zr–*D*), 1.58 (Zr–*D*), −0.41 (Zr–*D*). FTIR (KBr disc, cm^–1^): 2975 (s), 2954 (s), 2905 (s), 2861 (s), 1454 (m), 1382 (m), 1049 (m), 1020 (br, m), 915 (br, s), 812 (m).

### Pn*Ti(CH_2_SiMe_3_)_2_

A Youngs tap side arm Schlenk was charged with [Pn*TiCl](μ–Cl)_2_ (0.400 g, 0.656 mmol) and NaCH_2_SiMe_3_ (0.318 g, 2.885 mmol) to which pentane (40 mL) was added and allowed to stir for 16 h. The resulting purple suspension was filtered through celite on a sintered glass frit and eluted with pentane until colorless. The solution was reduced to minimum volume and cooled to −78 °C, which yielded a deep purple microcrystalline solid, which was collected in two crops in 72% total yield. ^1^H NMR (300 MHz, 298 K, C_6_D_6_) δ (ppm): 1.87 (s, 12H, 1,3,5,7-*Me*–Pn*), 1.84 (s, 6H, 2,6-*Me*–Pn*), 0.25 (s, 18H, Si*Me*_3_), −0.15 (s, 4H, Ti-C*H*_2_). ^13^C{^1^H} NMR (75 MHz, 298 K, C_6_D_6_) δ (ppm): 137.0 (4,8-Pn*), 130.1 (2,6-Pn*), 116.9 (1,3,5,7-Pn*), 56.4 (t, ^1^*J*_C–H_ = 105.3, Ti*C*H_2_), 13.2 (q, ^1^*J*_C–H_ = 126.8, 1,3,5,7-*Me*–Pn*), 11.6 (q, ^1^*J*_C–H_ = 127.2, 2,6-*Me*–Pn*}, 4.0 (q, ^1^*J*_C–H_ = 116.1, Si*Me_3_*). Anal calcd (found) for C_22_H_40_TiSi_2_: C, 64.47 (64.49); H, 9.87 (9.67).

### Pn*_3_Ti_3_(μ_2_-H)_3_(μ_3_-H)

Pn*Ti(CH_2_SiMe_3_)_2_ (0.150 g, 0.367 mmol) was dissolved in toluene (5 mL) and freeze–pump–thaw degassed. H_2_ gas (1 bar overpressure) was added at room temperature, leading to a slow color change from purple to green-black. The solution was stirred for 48 h before the volatiles were removed under dynamic vacuum overnight. Pn*_3_Ti_3_(μ_2_-H)_3_(μ_3_-H) was isolated as a pure black microcrystalline solid in 71% yield (0.061 mg, 0.086 mmol). ^1^H NMR (400 MHz, 298 K, C_6_D_6_) δ (ppm): 2.30 (s, 12H, 1,3,5,7-*Me*-Pn*), 2.15 (s, 6H, 2,6-*Me*-Pn*), −2.91 (s, 4H, Ti–*H*). ^13^C{^1^H} NMR (100 MHz, 298 K, C_6_D_6_) δ (ppm): 123.1 (4,8-Pn*), 122.0, 112.4 (1,3,5,7-Pn*), 15.5 (1,3,5,7-*Me*–Pn*), 12.1 (2,6-*Me*–Pn*). FTIR (KBr disc, cm^–1^): 2991 (w), 1959 (m), 2906 (s), 2857 (s), 1443 (br, s), 1373 (s), 1310 (w), 1042 (m), 1021 (s), 947 (m), 800 (m). Anal calcd (found) for C_42_H_58_Ti_3_: C, 71.40 (71.29); H, 8.27 (8.37).

### Pn*_3_Ti_3_(μ_2_-D)_3_(μ_3_-D)

Pn*Ti(CH_2_SiMe_3_)_2_ (0.150 g, 0.367 mmol) was dissolved in toluene (5 mL) and freeze–pump–thaw degassed. D_2_ gas (1 bar overpressure) was added at room temperature, leading to a slow color change from purple to green-black. The solution was stirred for 48 h before the volatiles were removed under dynamic vacuum overnight. Pn*_3_Ti_3_(μ_2_-D)_3_(μ_3_-D) was isolated as a pure black microcrystalline solid in 65% yield (0.057 g, 0.080 mmol). ^1^H NMR (400 MHz, 298 K, C_6_D_6_) δ (ppm): 2.30 (s, 12H, 1,3,5,7-*Me*–Pn*), 2.15 (s, 6H, 2,6-*Me*–Pn*). ^13^C{^1^H} NMR (100 MHz, 298 K, C_6_D_6_) δ (ppm): 123.8 (4,8-Pn*), 122.1, 112.2 (1,3,5,7-Pn*), 15.5 (1,3,5,7-*Me*–Pn*), 12.3 (2,6-*Me*–Pn*). ^2^D NMR (76.7 MHz, 298 K, C_6_D_6_,) δ (ppm): −3.1 ppm (Ti–*D*). Anal calcd (found) for C_42_H_54_D_4_Ti_3_: C, 71.00 (71.35); H, 8.79 (8.92).

## Supporting Information Available

Additional experimental details, representative NMR spectra, crystallographic collection and refinement details, and computational details (PDF)
