# Supplementary material for: Multimetallic Permethylpentalene Hydride Complexes
Source: Inorg Chem. 2022 Jul 25;61(31):12207–18. doi: 10.1021/acs.inorgchem.2c01267 (PMC9367693; doi:10.1021/acs.inorgchem.2c01267)
Supplement: Supplementary file 1 — ic2c01267_si_001.pdf [file ic2c01267_si_001.pdf]

Supporting Information

Multimetallic permethylpentalene hydride complexes

Duncan A. X. Fraser, Zoe R. Turner, Robert T. Cooper, Jean-Charles Buffet, Jennifer C. Green and Dermot O'Hare*.*

Chemistry Research Laboratory, Department of Chemistry, University of Oxford, Mansfield Road, Oxford OX1 3TA, United Kingdom. Tel: +44(0) 1865 285157; E-mail: dermot.ohare@chem.ox.ac.uk; jennifer.green@chem.ox.ac.uk

Table of Contents

I.	Representative NMR spectra	S2
II.	EPR Spectra	S3
III.	Crystallographic data	S4
IV.	Density functional theory calculation details	S11
V.	Extra NMR spectroscopic data	S12
VI.	References	S16

I. **Representative NMR spectra**

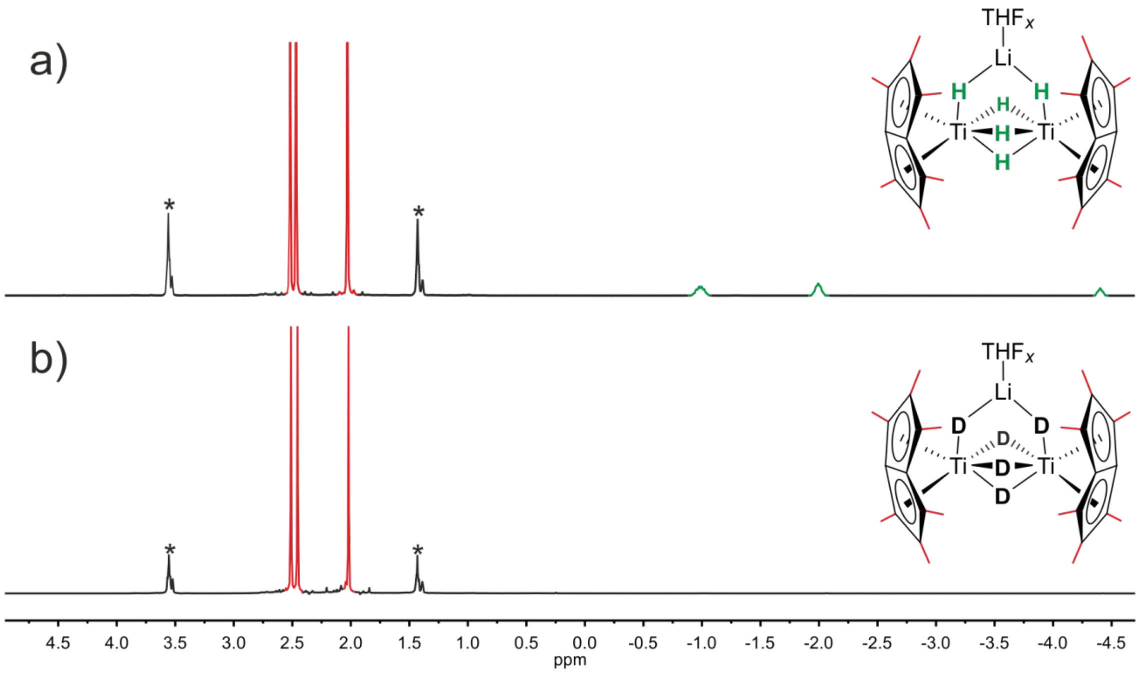

Figure S 1. ^{1}H NMR spectra (400 MHz, 298 K, C_6D_6/C_4D_8O 9:1) of a) $Pn^*{}_2Ti_2(\mu_2{-}H)_5Li.thf_x$ and b) $Pn^*{}_2Ti_2(\mu_2{-}D)_5Li.thf_x$. * = THF residual from synthesis.

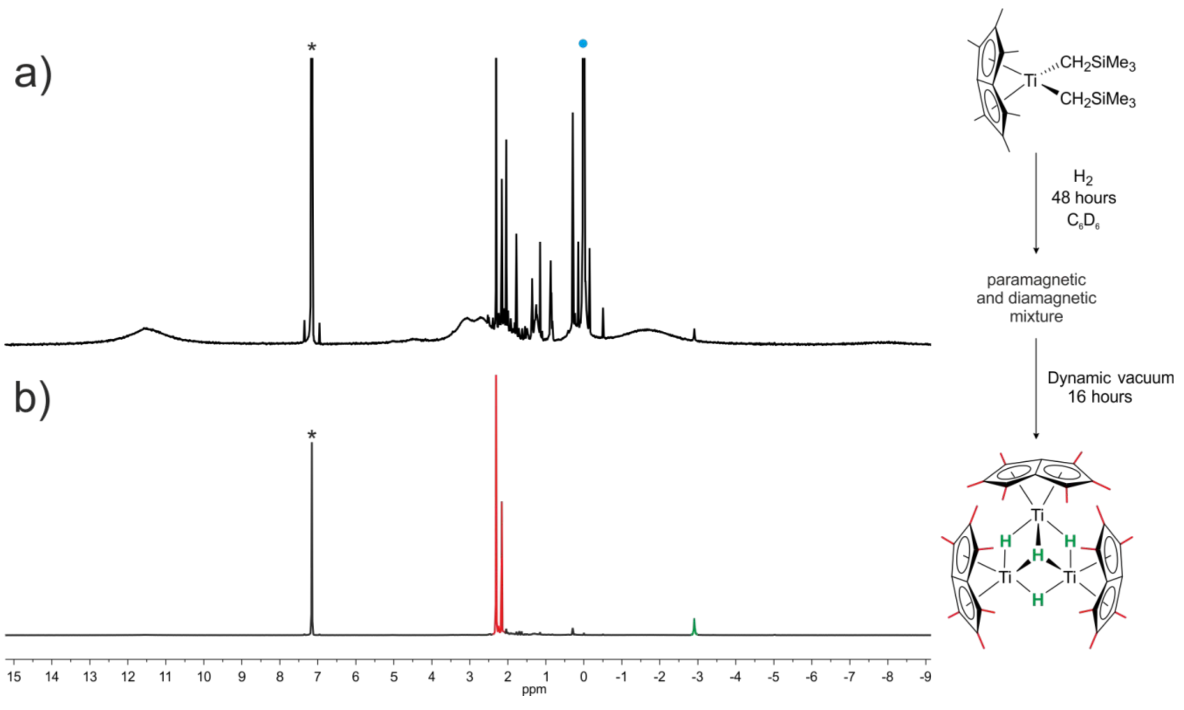

Figure S 2. ^{1}H NMR spectra (400 MHz, 298 K, C_6D_6) of a) $Pn^*Ti(CH_2SiMe_3)_2$ under 1 bar H_2 and b) the same sample after exposure to dynamic vacuum for 16 hours. * = C_6D_6 solvent residual signals, blue circle = $SiMe_4$.

II. **EPR spectra**

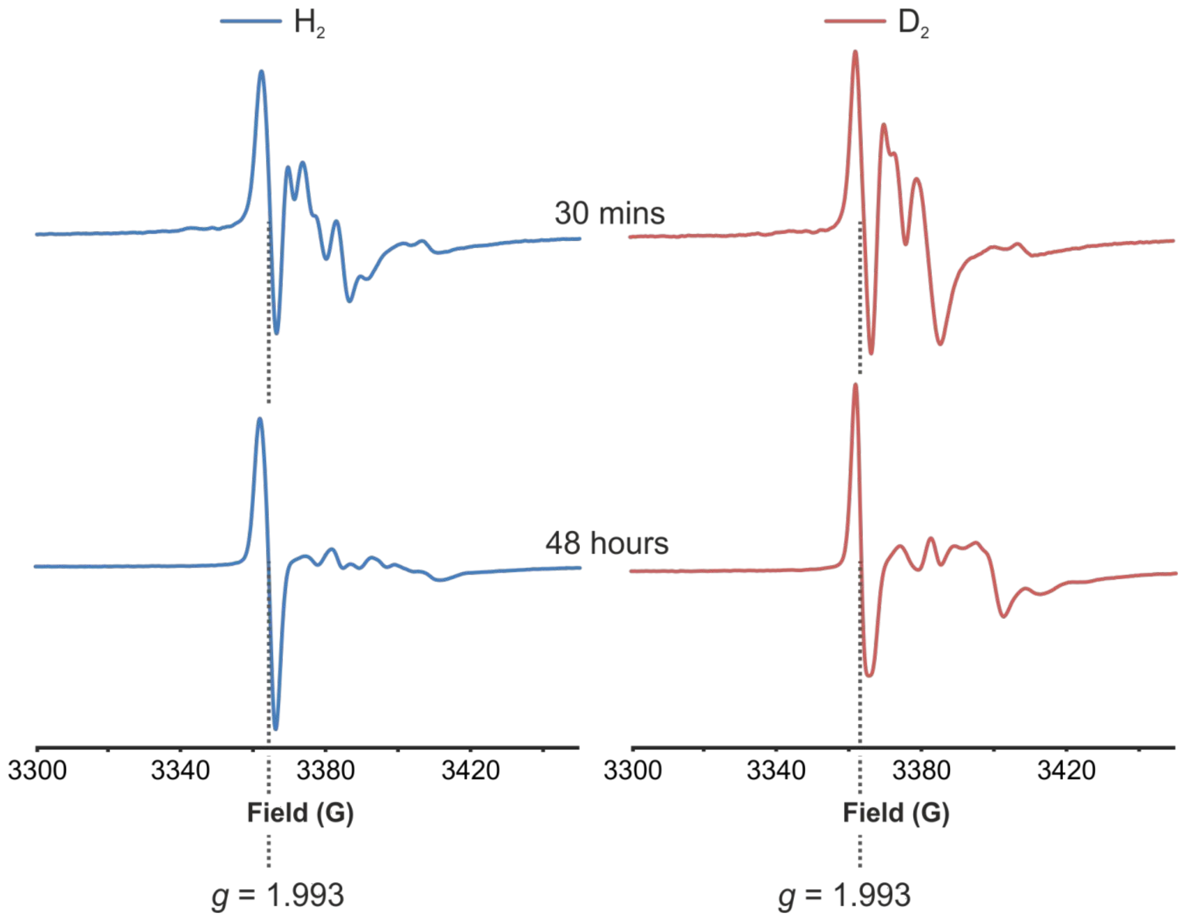

Figure S3. X-band EPR spectra (5 mM toluene solution, 298 K) of $Pn^*_3Ti_3(\mu_2\text{-}H)_3(\mu_3\text{-}H)$ and $Pn^*_3Ti_3(\mu_2\text{-}D)_3(\mu_3\text{-}D)$ under H_2 and D_2 gas (1 bar overpressure).

III. Crystallographic data

Crystals were mounted on MiTeGen MicroMounts using perfluoropolyether oil and rapidly transferred to a goniometer head on a diffractometer fitted with an Oxford Cryosystems Cryostream open-flow nitrogen cooling device.[1] Data collections were carried out at 150 K using an Oxford Diffraction Supernova diffractometer using mirror monochromated Cu Kα radiation (λ = 1.54178 Å) and data were processed using CrysalisPro.[2] The structures were solved using direct methods (SIR-92)[3] or a charge flipping algorithm (SUPERFLIP) and refined by full-matrix least-squares procedures using the Win-GX software suite.[4] Solid state structural data have been deposited in the Cambridge Crystallographic Data Centre (2164608–2164612).

Table S 1. Selected experimental crystallographic data.

Complex	**$Pn^*_2Ti_2(\mu_2–H)_5Li.thf_2$**	**$Pn^*_2Ti_2(\mu_2–H)_5Li.thf$**	**$[Pn^*_2Ti_2H_5Li.dioxane]_n$**
**Crystal data**			
Chemical formula	$C_{36}H_{57}LiO_2Ti_2$	$C_{32}H_{49}LiOTi_2·0.5(C_6H_6)$	$C_{64}H_{98}Li_2O_4Ti_4·4(C_6H_6)$
M_r	624.55	591.50	1449.33
Crystal system, space group	Monoclinic, $P2_1/n$	Monoclinic, $P2_1/n$	Triclinic, $P\overline{1}$
Temperature (K)	150	150	150
a, *b*, *c* (Å)	10.1320 (3), 18.6943 (5), 19.0992 (4)	15.6458 (3), 11.4721 (3), 18.4284 (4)	10.2881 (2), 19.3994 (3), 21.0051 (3)
α, β, γ (°)	90, 103.953 (3), 90	90, 96.835 (2) 90	98.038 (1), 91.627 (1), 99.117 (1)
V (Å^3)	3510.85 (16)	3284.21 (13)	4093.15 (12)
Z	4	4	2
Radiation type	Cu $K\alpha$	Cu $K\alpha$	Cu $K\alpha$
μ (mm^{-1})	4.07	4.30	3.56
Crystal size (mm)	0.20 × 0.12 × 0.07	0.18 × 0.14 × 0.10	0.34 × 0.10 × 0.08
**Data Collection**			
Diffractometer	SuperNova, Dual, Cu at zero, Atlas diffractometer	SuperNova, Dual, Cu at zero, Atlas diffractometer	SuperNova, Dual, Cu at zero, Atlas diffractometer
Absorption correction	Multi-scan *CrysAlis PRO* 1.171.38.41 (Rigaku Oxford Diffraction, 2015) Empirical absorption correction using spherical harmonics, implemented in SCALE3 ABSPACK scaling algorithm.	Multi-scan *CrysAlis PRO* 1.171.38.41 (Rigaku Oxford Diffraction, 2015) Empirical absorption correction using spherical harmonics, implemented in SCALE3 ABSPACK scaling algorithm.	Multi-scan *CrysAlis PRO* 1.171.38.41 (Rigaku Oxford Diffraction, 2015) Empirical absorption correction using spherical harmonics, implemented in SCALE3 ABSPACK scaling algorithm.

T_{min}, T_{max}	0.666, 1.000	0.785, 1.000	0.604, 1.000
No. of measured, independent and observed [$I > 2\sigma(I)$] reflections	73094, 7196, 6456	20931, 6709, 6013	83587, 16716, 14669
R_{int}	0.089	0.022	0.056
	**Refinement**		
$R[F^2 > 2\sigma(F^2)]$, $wR(F^2)$, S	0.043, 0.113, 1.04	0.031, 0.087, 1.05	0.045, 0.125, 0.99
No. of reflections	7196	6709	16716
No. of parameters	476	421	978
No. of restraints	14	0	0
$(\Delta/\sigma)_{max}$	0.001	0.002	0.002
$\Delta\rho_{max}$, $\Delta\rho_{min}$ (e Å^{-3})	0.40, -0.61	0.32, -0.40	0.42, -0.35

Complex	**[Pn*$_2$Zr$_2$(μ$_2$–H)$_4$(μ$_3$–H)Li.thf]$_2$**	**Pn*$_3$Ti$_3$(μ$_2$–H)$_3$(μ$_3$–H)**
	**Crystal data**	
Chemical formula	$C_{64}H_{98}Li_2O_2Zr_4 \cdot C_6H_6$	$C_{45}H_{61}Ti_3$
M_r	1356.29	745.63
Crystal system, space group	Monoclinic, $P2_1/n$	Monoclinic, $C2/c$
Temperature (K)	150	150
a, b, c (Å)	12.0214 (1), 13.1350 (1), 21.1876 (1)	10.9887 (2), 20.3156 (3), 37.9260 (5)
α, β, γ (°)	90, 93.192 (1), 90	90, 91.315 (1), 90

V (Å^3)	3340.35 (4)	8464.4 (2)
Z	2	8
Radiation type	Cu $K\alpha$	Cu $K\alpha$
μ (mm^{-1})	5.28	4.89
Crystal size (mm)	0.19 × 0.13 × 0.10	0.24 × 0.13 × 0.10
	**Data Collection**	
Diffractometer	SuperNova, Dual, Cu at zero, Atlas diffractometer	SuperNova, Dual, Cu at zero, Atlas diffractometer
Absorption correction	Multi-scan *CrysAlis PRO* 1.171.38.41 (Rigaku Oxford Diffraction, 2015) Empirical absorption correction using spherical harmonics, implemented in SCALE3 ABSPACK scaling algorithm.	Multi-scan *CrysAlis PRO* 1.171.38.41 (Rigaku Oxford Diffraction, 2015) Empirical absorption correction using spherical harmonics, implemented in SCALE3 ABSPACK scaling algorithm.
T_{min}, T_{max}	0.759, 1.000	0.902, 0.954
No. of measured, independent and observed [$I > 2\sigma(I)$] reflections	34327, 6824, 6660	40601, 8671, 8306
R_{int}	0.017	0.026
	**Refinement**	
$R[F^2 > 2\sigma(F^2)]$, $wR(F^2)$, S	0.019, 0.048, 1.08	0.034, 0.086, 1.11
No. of reflections	6824	8671
No. of parameters	421	467
No. of restraints	0	0

$(\Delta/\sigma)_{max}$	0.003	0.004
$\Delta\rho_{max}$, $\Delta\rho_{min}$ (e Å^{-3})	0.31, -0.49	0.71, -0.24

Computer programs: *CrysAlis PRO* 1.171.39.46 (Rigaku Oxford Diffraction, 2018) *CrysAlis PRO*, Agilent Technologies, Version 1.171.35.21 (release 20-01-2012 CrysAlis171 .NET) (compiled Jan 23 2012,18:06:46), : SUPERFLIP. Palatinus, L.; Chapuis, G. J. Appl. Cryst. 2007, 40, 786-790, : Sir-92. Altomare, A.; Cascarano, G.; Giacovazzo, C.; Guagliardi, A. J. Appl. Cryst. 1994, 27, 435., *SIR92* Altomare, A.; Cascarano, G.; Giacovazzo, C.; Guagliardi, A. J. Appl. Cryst. 1994, 27, 435, *SHELXL2014* (Sheldrick, 2014), *ORTEP-3 for Windows*. Farrugia, L. J. J. Appl. Cryst. 1997, 30, 565.

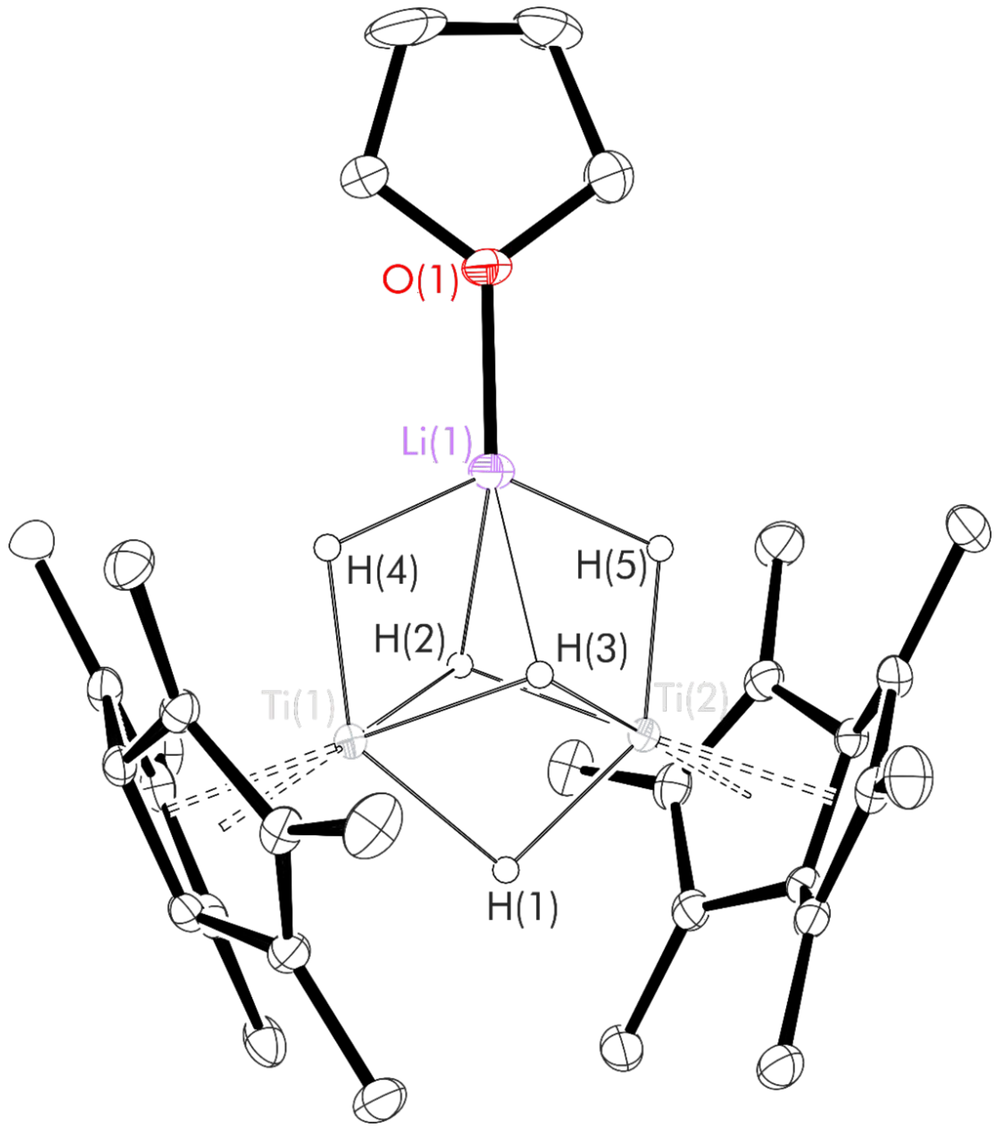

Figure S 4. Thermal displacement ellipsoid drawings (30% probability) of $Pn^*_2Ti_2(\mu_2\text{–}H)_5Li$.thf. All hydrogen atoms expect for H(1)–H(5), and benzene solvent of crystallisation have been omitted for clarity.

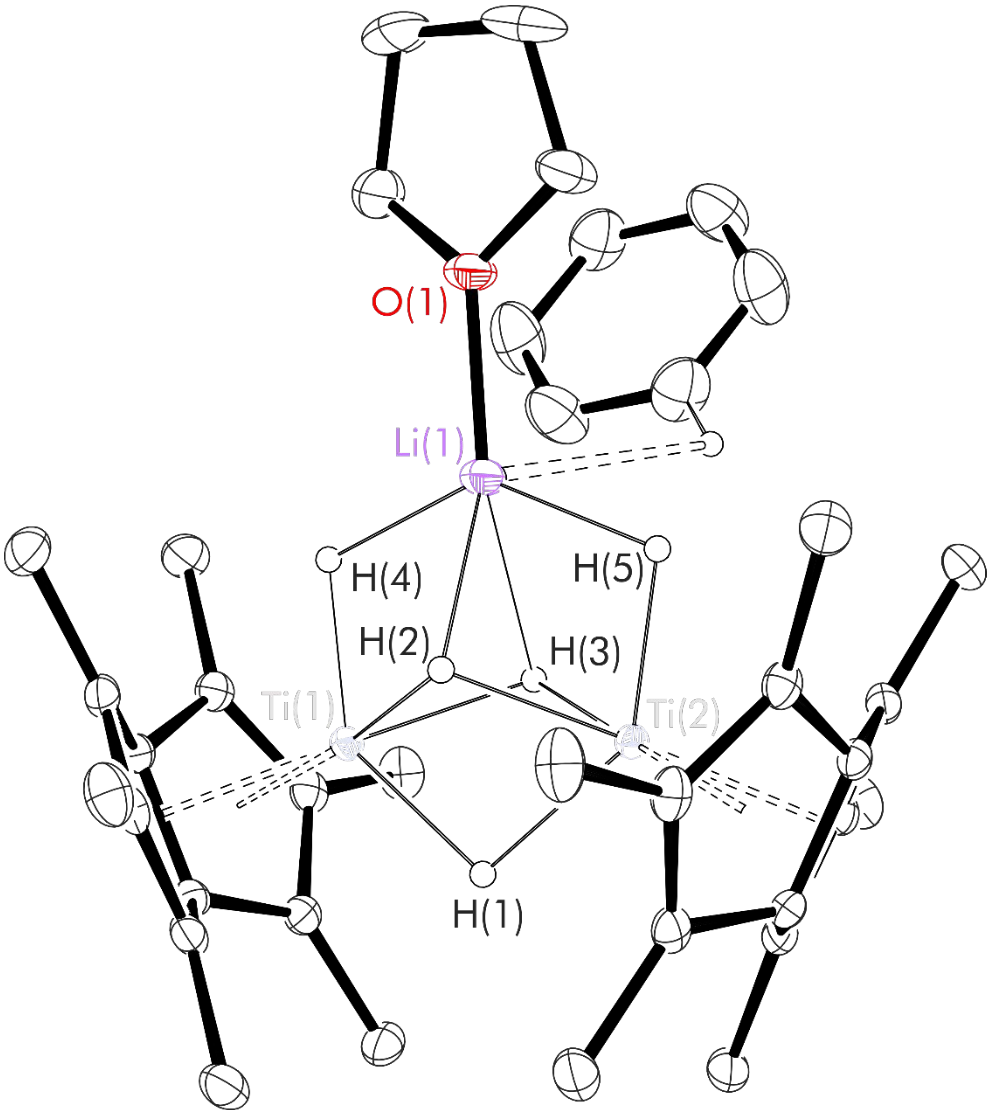

Figure S 5. Thermal displacement ellipsoid drawings (30% probability) of $Pn^*_2Ti_2(\mu_2\text{–}H)_5Li.thf\cdot C_6H_6$. All hydrogen atoms expect for H(1)–H(5) have been omitted for clarity. The benzene solvent of crystallisation is included to indicate the close contact with Li(1).

IV. **Density functional theory calculation details**

Density Functional Theory calculations were performed by Prof. Jenny Green ($Pn^*_3Ti_3(\mu_2–H)_3(\mu_3–H)$) or Mr Duncan Fraser. With the exception of $Pn^*_3Ti_3(\mu_2–H)_3(\mu_3–H)$, calculations were performed using the Orca software package.[5] Geometry optimisations and single-point calculations on the optimised geometries were carried out at the BP86 [6, 7] or B3LYP[7-9] level of theory. Unless otherwise stated Ahlrich's split-valence basis set, def2–SVP was used for elements up to carbon, while the triple-zeta basis set, def2-TZVP(-f) was used for heavier elements.[10-12] The RI approximation was used in all cases to accelerate the calculations. [13-15] Orbitals were generated with the IBOVIEW software.[16, 17] For $Pn^*_3Ti_3(\mu_2–H)_3(\mu_3–H)$, density functional calculations were carried using the Amsterdam Density Functional package[18] and triple-ζ quality basis sets augmented with a one polarisation function (ADF basis TZP). The local density approximation (LDA) of Vosko, Wilk and Nusair (VWN) [19] was used together with the exchange correlation corrections of Becke and Perdew (BP86).[6, 7]

V Additional NMR spectroscopic data

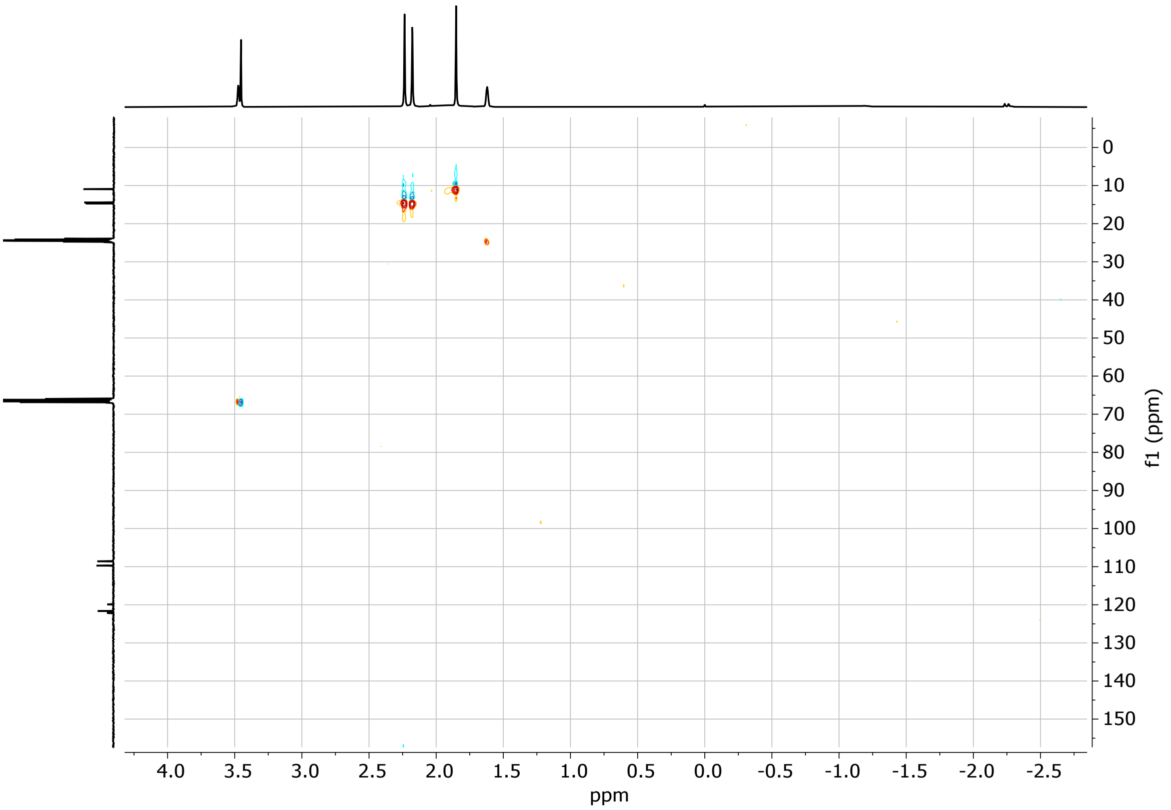

Figure S6. HSBC NMR spectrum (125 MHz, 298 K, C_6D_6/C_4D_8O 9:1) of $Pn^*{}_2Ti_2(\mu_2{-}H)_5Li.thf_x$.

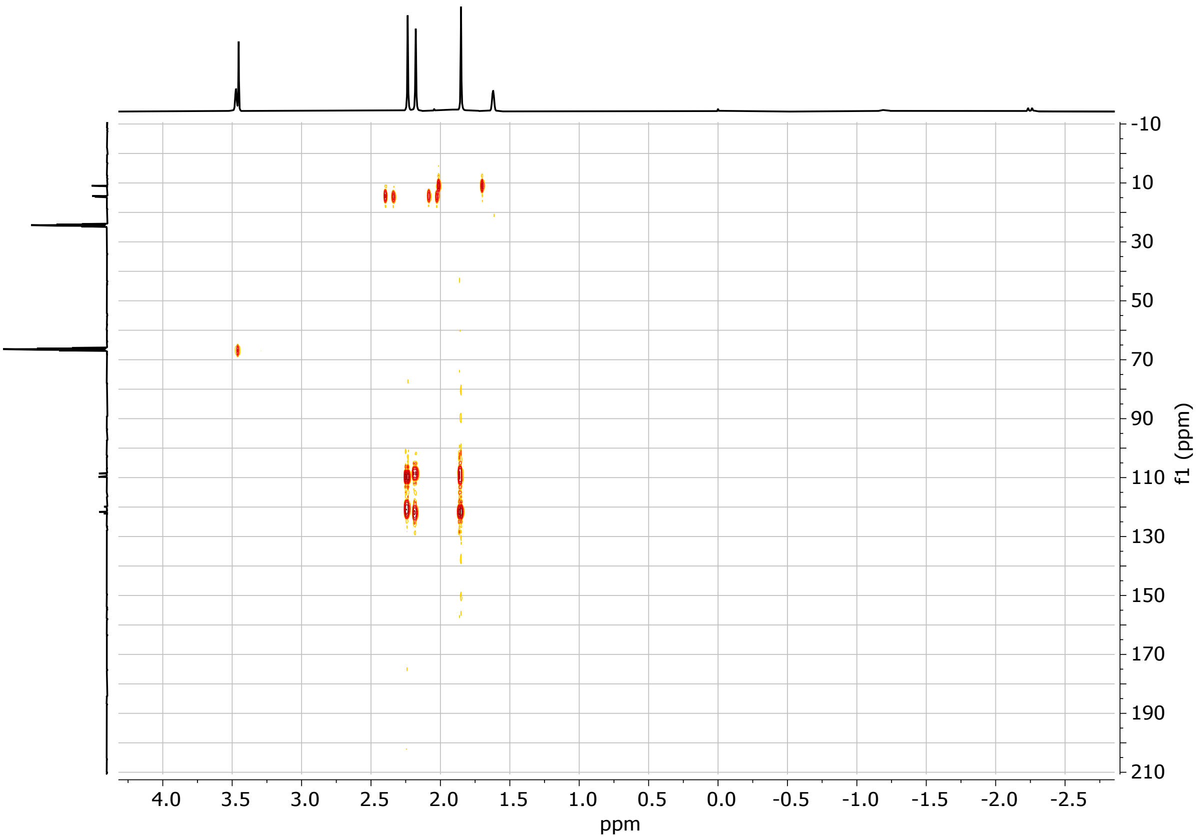

Figure S7. HMBC NMR spectrum (125 MHz, 298 K, C_6D_6/C_4D_8O 9:1) of $Pn^*{}_2Ti_2(\mu_2{-}H)_5Li.thf_x$.

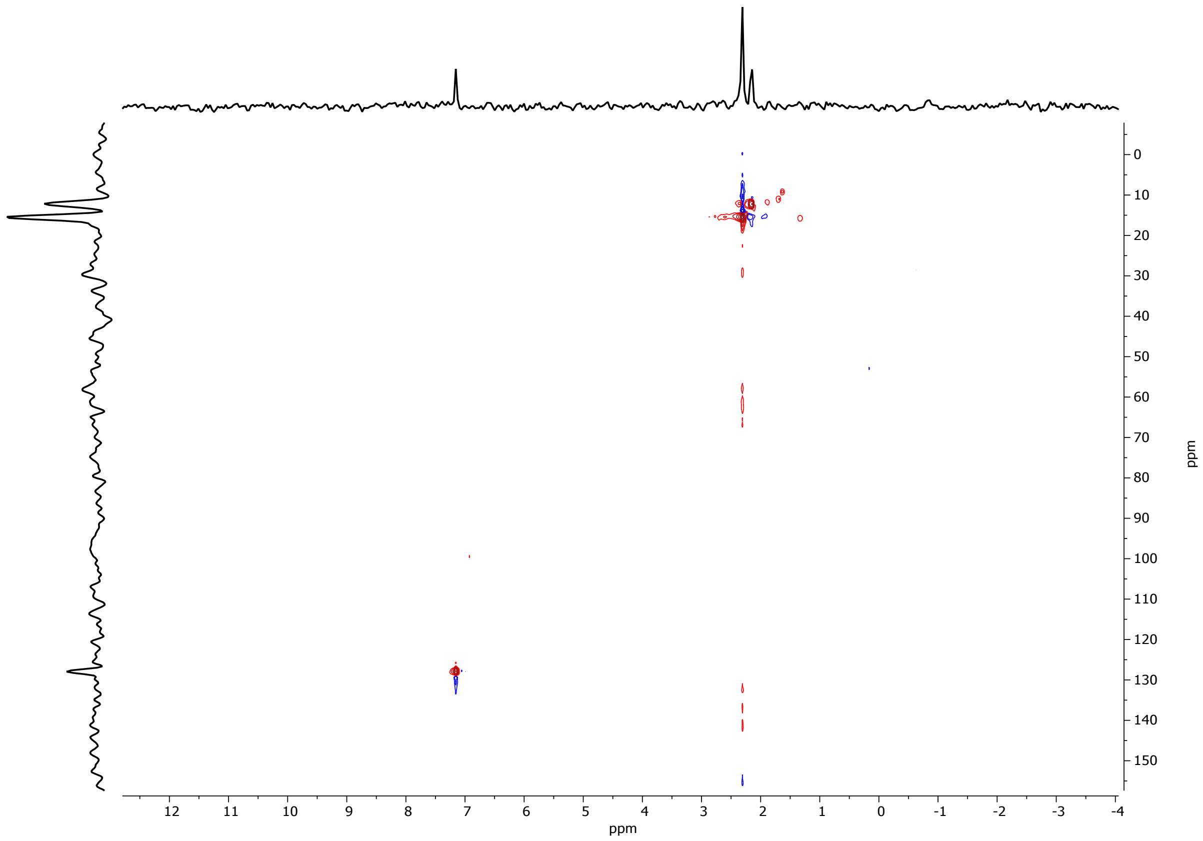

Figure S8. HSQC NMR spectrum (125 MHz, 298 K, C_6D_6) of $Pn^*_3Ti_3(\mu_2\text{-}H)_3(\mu_3\text{-}H)$.

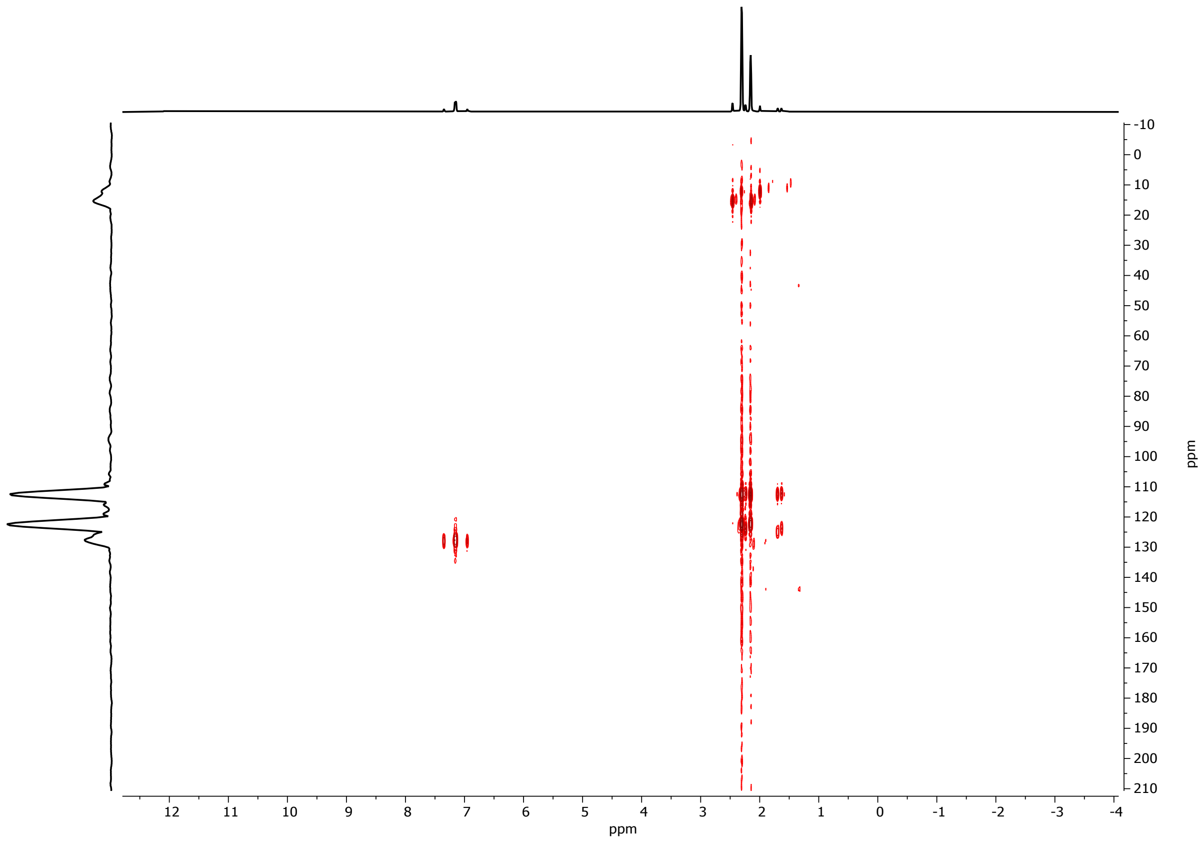

Figure S9. HMBC NMR spectrum (125 MHz, 298 K, C_6D_6) of $Pn^*_3Ti_3(\mu_2\text{-}H)_3(\mu_3\text{-}H)$.

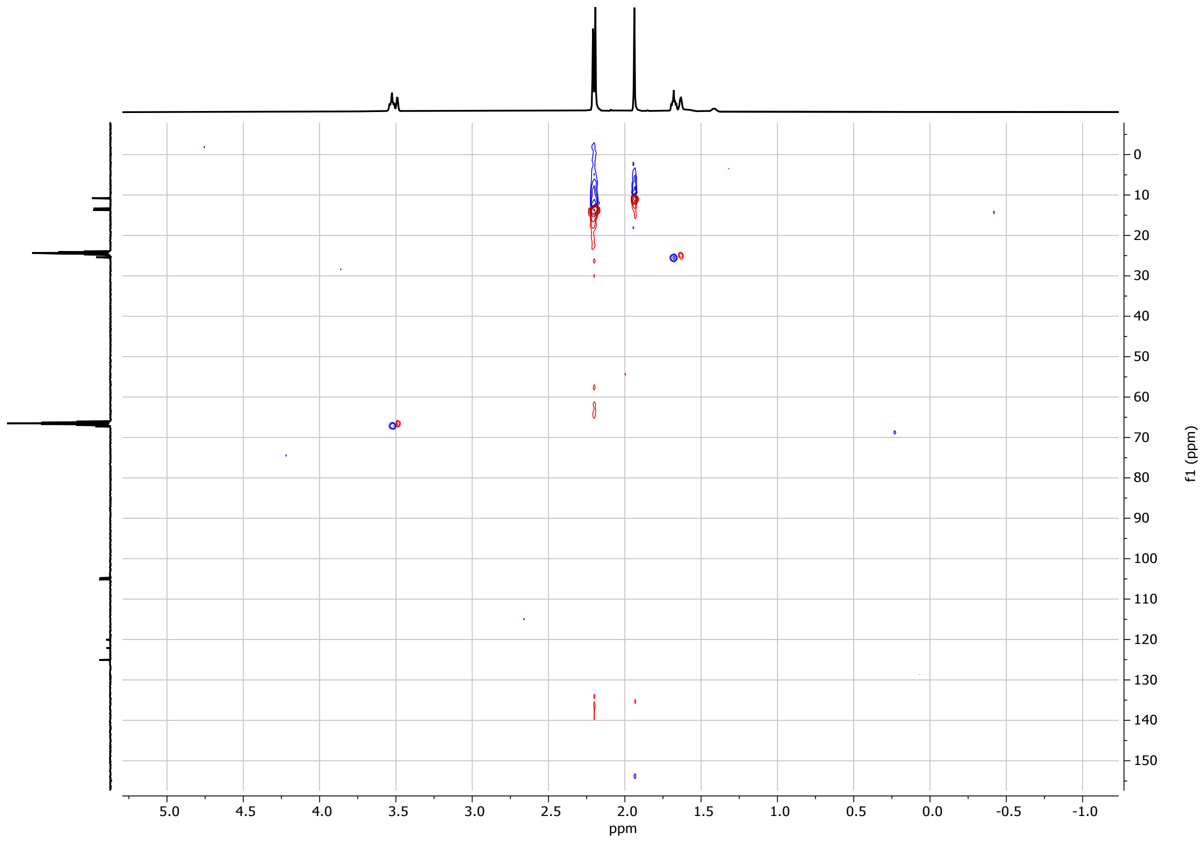

Figure S10. HSQC NMR spectrum (125 MHz, 298 K, C_6D_6) of [Pn*$_2$Zr$_2$(μ_2–H)$_4$(μ_3–H)Li.thf]$_2$.

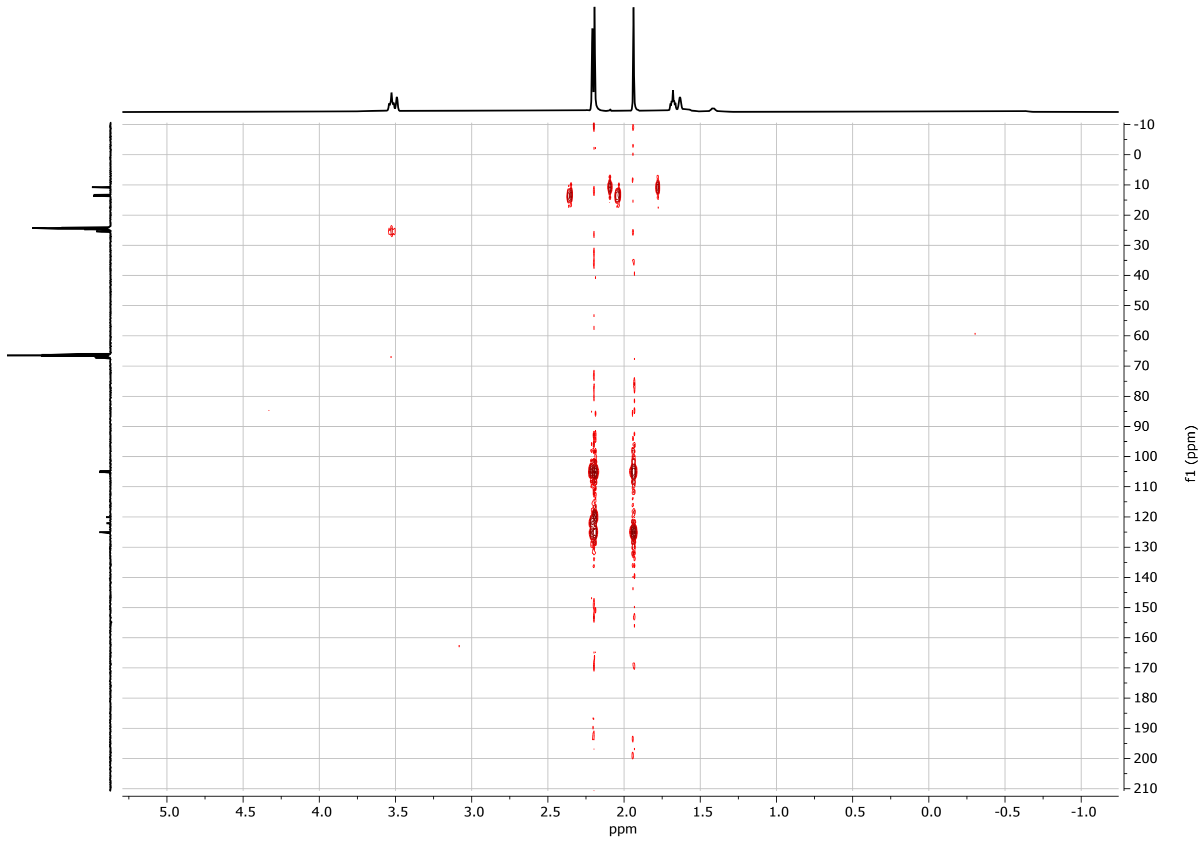

Figure S11. HMBC NMR spectrum (125 MHz, 298 K, C_6D_6) of [Pn*$_2$Zr$_2$(μ_2–H)$_4$(μ_3–H)Li.thf]$_2$.

VI References

1. Cosier, J.; Glazer, A. M., A nitrogen-gas-stream cryostat for general X-ray diffraction studies. *J. Appl. Crystallogr.* **1986,** *19* (2), 105-107.
2. CrysAlisPRO, Oxford Diffraction /Agilent Technologies UK Ltd, Yarnton, England.
3. Altomare, A.; Cascarano, G.; Giacovazzo, C.; Guagliardi, A.; Burla, M. C.; Polidori, G.; Camalli, M., SIR92 - a program for automatic solution of crystal structures by direct methods. *J. Appl. Crystallogr.* **1994,** *27* (3), 435.
4. Palatinus, L.; Chapuis, G., SUPERFLIP - a computer program for the solution of crystal structures by charge flipping in arbitrary dimensions. *J. Appl. Crystallogr.* **2007,** *40* (4), 786-790.
5. Neese, F., The ORCA program system. *Wiley Interdisciplinary Reviews: Computational Molecular Science* **2012,** *2* (1), 73-78.
6. Becke, A. D., Density-functional exchange-energy approximation with correct asymptotic behavior. *Physical Review A* **1988,** *38* (6), 3098-3100.
7. Perdew, J. P., Density-functional approximation for the correlation energy of the inhomogeneous electron gas. *Phys. Rev. B: Condens. Matter Mater. Phys.* **1986,** *33* (12), 8822-8824.
8. Becke, A. D., Density functional calculations of molecular bond energies. *J. Chem. Phys.* **1986,** *84* (8), 4524-4529.
9. Perdew, J. P.; Yue, W., Accurate and simple density functional for the electronic exchange energy: Generalized gradient approximation. *Phys. Rev. B: Condens. Matter Mater. Phys.* **1986,** *33* (12), 8800-8802.
10. Schäfer, A.; Horn, H.; Ahlrichs, R., Fully optimized contracted Gaussian basis sets for atoms Li to Kr. **1992,** *97* (4), 2571-2577.
11. Schäfer, A.; Huber, C.; Ahlrichs, R., Fully optimized contracted Gaussian basis sets of triple zeta valence quality for atoms Li to Kr. **1994,** *100* (8), 5829-5835.
12. Weigend, F.; Ahlrichs, R., Balanced basis sets of split valence, triple zeta valence and quadruple zeta valence quality for H to Rn: Design and assessment of accuracy. *PCCP* **2005,** *7* (18), 3297-3305.
13. Eichkorn, K.; Treutler, O.; Öhm, H.; Häser, M.; Ahlrichs, R., Auxiliary basis sets to approximate Coulomb potentials (Chem. Phys. Letters 240 (1995) 283-290). *Chem. Phys. Lett.* **1995,** *242* (6), 652-660.
14. Eichkorn, K.; Weigend, F.; Treutler, O.; Ahlrichs, R., Auxiliary basis sets for main row atoms and transition metals and their use to approximate Coulomb potentials. *Theor. Chem. Acc.* **1997,** *97* (1-4), 119-124.
15. Eichkorn, K.; Treutler, O.; Öhm, H.; Häser, M.; Ahlrichs, R., Auxiliary basis sets to approximate Coulomb potentials. *Chem. Phys. Lett.* **1995,** *240* (4), 283-290.
16. Knizia, G., Intrinsic Atomic Orbitals: An Unbiased Bridge between Quantum Theory and Chemical Concepts. *J. Chem. Theory Comput.* **2013,** *9* (11), 4834-4843.
17. Knizia, G.; Klein, J. E. M. N., Electron Flow in Reaction Mechanisms—Revealed from First Principles. **2015,** *54* (18), 5518-5522.
18. te Velde, G.; Bickelhaupt, F. M.; Baerends, E. J.; Fonseca Guerra, C.; van Gisbergen, S. J. A.; Snijders, J. G.; Ziegler, T., Chemistry with ADF. **2001,** *22* (9), 931-967.
19. Vosko, S. H.; Wilk, L.; Nusair, M., Accurate spin-dependent electron liquid correlation energies for local spin density calculations: a critical analysis. **1980,** *58* (8), 1200-1211.
